# Exploring Carbamazepine Polymorph Crystal Growth in Water by Enhanced Sampling Simulations

**DOI:** 10.1021/acsomega.4c05458

**Published:** 2024-08-16

**Authors:** Radost Herboth, Alexander P. Lyubartsev

**Affiliations:** Department of Materials and Environmental Chemistry, Stockholm University, Svante Arrhenius väg 16C, 106 91 Stockholm, Sweden

## Abstract

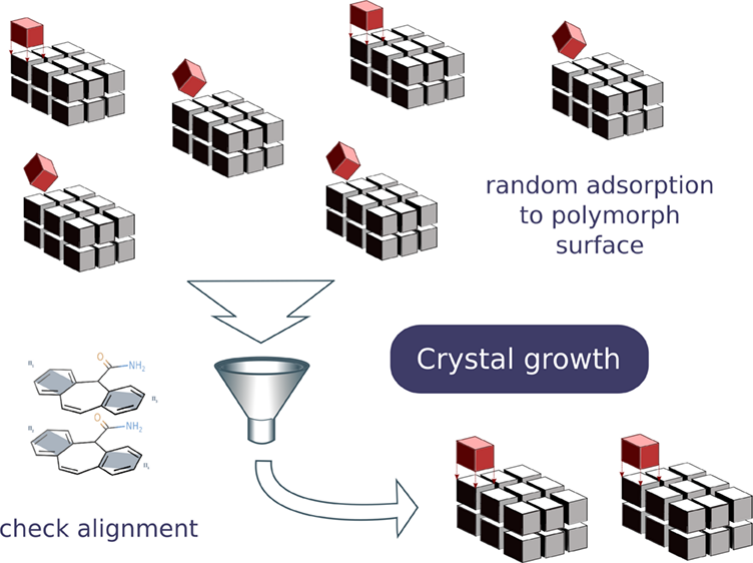

In this work, the polymorphism of the active pharmaceutical ingredient carbamazepine (CBZ) was investigated by using molecular dynamics simulations with an enhanced sampling scheme. A single molecule of CBZ attaching to flat surfaces of different polymorphs was used as a model for secondary nucleation in water. A novel approach was developed to compute the free energy profile characterizing the adsorption of molecules with orientation aligned with the crystal structure of the surface. The distribution of states that showed alignment was used to rescale the adsorption free energy to include only the contribution that is consistent with crystal growth. The resulting free energy surfaces showed favorable thermodynamics for the most stable form, Form III and the second most stable form, Form I. The primary crystallization product, a dihydrate, was found to be less favorable, implying a nonclassical crystallization pathway. We suggest that a major contribution determining the energetics is the hydrophobicity of the surface. This thermodynamic ranking provides valuable information about the molecular pathways of polymorph growth and will further contribute to the understanding of the crystallization process of CBZ, which is imperative since polymorph formation can alter the physical properties of a drug significantly.

## Introduction

Polymorphism, the ability of a material to crystallize in different solid forms, is highly common and mostly unpredictable based on molecular structure.^1^ This poses a challenge, especially in the manufacturing of effective drugs, where active pharmaceutical ingredients (APIs) may crystallize in polymorphic forms that differ greatly in their physical and chemical properties, like solubility and bioavailability.^2^ The polymorphism of many APIs is known, and the various structures have been characterized and studied extensively. However, there is still a lack of feasible methods for directed crystallization of the desired forms, as this requires an in-depth understanding of the factors that drive crystallization to different polymorphs.

**Figure 1:**
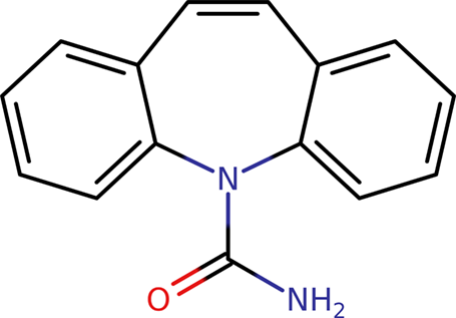
API carbamazepine (CBZ).

Carbamazepine (Figure 1), often abbreviated as CBZ, has long been the workhorse of polymorphism studies of all kinds. CBZ is a well-established drug in the treatment of epilepsy^3^ and trigeminal neuralgia,^4^ which operates through the inhibition of voltage-gated sodium channels.^5^ Its polymorphism has been thoroughly investigated, and four stable anhydrous forms of the molecule are known: A P-monoclinic form known as Form III,^6−8^ a trigonal form known as Form II,^9^ a triclinic form known as Form I,^10^ and a C-monoclinic form known as Form IV.^11^ Melting points differ noticeably between polymorphs and phase transitions between the forms upon heating have been reported.^12^ The most stable polymorph is Form III, which is also most commonly found in conventional tablets.^13^ CBZ is an example of packing polymorphism, meaning that it is only the arrangement of the fundamental units in space that differs between the polymorphs. In the four anhydrous polymorphs, this unit is a hydrogen-bonded dimer, where the amide groups of two CBZ molecules show a *R*_2_^2^(8) hydrogen-bonding motif. Recently a fifth anhydrous form named Form V has been reported, which shows catemeric hydrogen bonds,^14^ but its stability has not been investigated.

All forms except Form I can be prepared from a solution in ethanol; however, Form IV only crystallizes in the presence of hydroxypropylcellulose in this solvent. Form I is only obtained at high temperatures, e.g., by heating Form III for several hours.^10^ In aqueous solutions, the most stable form is that of a dihydrate and Forms I and III have been reported to convert to the dihydrate upon contact with water.^15^ The morphology of Forms I and II, as well as the dihydrate, has been described as needle-like,^10,16^ while Form III is observed as prismatic^17^ and Form IV as plate-like particles.^11^ The choice of solvent and its influence on the nucleation of CBZ has been investigated for a few polymorphs,^18^ but no systematic studies have been conducted so far.

CBZ is generally poorly soluble in water, but the dihydrate form has been reported to reduce the dissolution rate further.^19^ This has led to problems with storage under increased moisture conditions, which was speculated to be the cause of several reports of clinical failures that led to a temporary withdrawal of the generic tablet from the market in the late 1980s.^20,21^

Since crystallization is a molecular-scale process and as such hard to probe using experimental techniques, it seems sensible to complement the existing data with computational methods such as molecular dynamics (MD)-based simulations that can capture the system down to the atomistic level. It is common in crystallization studies to distinguish between primary nucleation, where crystalline nuclei arise spontaneously from solution, and secondary nucleation, where a crystalline nucleus is already present and solute molecules attach to it to grow the cluster. Both processes have been described by MD simulations, but generally, the time scales of primary nucleation lie far beyond what can be simulated at the present moment, as many of the molecular events are scarce and require extensive sampling. Hence, enhanced sampling methods have been the focus of many of the studies that have been conducted on primary nucleation and polymorph formation.^22^ Many of them rely on the design of a suitable collective variable (CV) that allows the simulation to proceed along the reaction path while capturing all relevant states as distinct from each other. Such CVs can be based on the symmetry of the emerging cluster, molecular ordering parameters such as local crystallinity or pair correlation functions or physical properties like enthalpy/entropy or the structure factor of the crystal and can be reduced in their dimensionality for better computational performance using methods like linear discriminant analysis or machine-learning protocols.^23^ The primary nucleation of urea as a model for organic molecules has been studied extensively by Salvalaglio and co-workers using a local crystallinity CV.^24−26^ Other works have focused on the formation of prenucleation clusters, for example in 1,3,5-tris(4-bromophenyl)benzene,^27^*p*-aminobenzoic acid (pABA),^28^ or paracetamol.^29^

Secondary nucleation (and the related process dissolution) has been explored using classical MD,^30^ metadynamics,^31,32^ and new methods like constant chemical potential MD (CμMD), which has been successfully applied to the study of ionic crystals^33^ as well as organic molecules such as urea,^34,35^ isoniazid,^36^ and naphthalene.^37^ It is common to also use CVs as a measure of crystallinity in the analysis of the trajectories, even if they are not directly used in the simulations.

Nevertheless, most of the methods/CVs applied to the direct study of primary and secondary nucleation have only been tested for ionic crystals or small (rigid) organic molecules that show no polymorphism. CBZ possesses a fair amount of conformational flexibility, and packing polymorphs present an additional challenge in the design of a suitable CV that would be able to distinguish both disordered states and the different anhydrous forms and the dihydrate but also be low-dimensional so as not to increase computational cost. Neither primary nor secondary nucleation has yet been observed directly in simulations for this molecule. Gadelmeier et al.^38^ used the patterns of molecular packing as a jumping-off point in the computational comparison of the four anhydrous polymorphs. Forms III and IV show a so-called sandwich-herringbone configuration of the aromatic ring systems, while Forms I and II show a stacked, slightly offset configuration. This leads to Forms I and II showing a more delicate balance between nonpolar interactions and hydrogen bonding, while Forms III and IV are dominated by the interactions between the stacked aromatic rings. However, they concluded that it was not feasible to simply manipulate the crystallization on the basis of these patterns. Instead, they hypothesize that size effects play a role in the stability of each polymorph.

Here we aim to study the secondary nucleation of CBZ, where a crystal nucleus of a certain form is already present in solution, and solutes are attaching and detaching several times within the simulation. For this, a one-dimensional metadynamics approach was chosen with water as the polar solvent of choice. Metadynamics samples the states along the reaction path by accumulating a history-dependent bias that is deposited at each step of the simulation. This ultimately leads to the free energy surface being filled and the whole CV space being sampled.^39^ In principle, this type of study could also be done using other forms of enhanced sampling, but metadynamics offers some advantages. First, the way of sampling is closer to the real-life process than in an alchemical transformation (such as in free energy perturbation methods) of the system, allowing the solute and solvent to interact naturally along the given pathway with the solute repeatedly adsorbing/desorbing at the surface. It also requires less preliminary knowledge of the pathway and the binding site as opposed to methods such as umbrella sampling. The system is simplified to be a single molecule attached to a perfect surface of a given polymorphic form. A first question that arises when looking at the crystal growth of the anhydrous CBZ polymorphs is what the unit is that attaches to a crystal nucleus. A computational study focused on CBZ crystallization precursors and the effect of the choice of solvents on their stability^40^ found that nonpolar solvents favor a hydrogen-bonded dimer, while polar solvents promote dimers stabilized by the π-stacking interactions of the ring system, which has also been observed experimentally.^41^ Since all polymorphs show the same fundamental unit, a hydrogen-bonded dimer (cf. Figure S1a), it would be possible that this is also the crystal growth unit. However, that would preclude that the unit is stable in the solvent, which is not expected for water as a polar solvent and which we confirmed in preliminary simulations (cf. Figure S2). One could opt to use the π-stacked dimer instead (cf. Figure S1b,c), but since this type of stacking only occurs in Forms III and IV, but not in Forms I and II, it was decided to keep the simulations uniform and only use a single molecule.

This approach shares some similarities with the concept of attachment energy (*E*_att_) calculations, which are routinely used to predict the crystal habit of compounds. In the original approach by Hartman and Bennema, only the interaction of a flat surface with a new growth layer is considered on a chosen reference molecule.^42^ The resulting interaction energy *E*_att_ can be used to construct the equilibrium morphology of the crystal, similar to a Wulff construction. This is in principle done in a vacuum, but solvent effects can be included by taking into account the adsorption energy of the solvent or the polarity differences between the surface and the solvent.^43^ However, most crystals are not crystallized in their equilibrium habit, since external factors and solvent kinetics play a large role in the stabilization of the surfaces.^44^ Moreover, there is no description of entropy included in the model, but it is known that the orientation of adsorbing molecules as well as competing adsorbents can have a large impact on the crystal habit.^45^ In contrast, the description of attachment by enhanced sampling explicitly includes solvent adsorption and desorption as well as the desolvation of the solute and its competition with the solvent molecules at the surface. Temperature is controlled independently and at a more realistic value for crystallization, as attachment energies are effectively calculated at 0 K. Lastly, since we are computing free energies and not pure interaction energies, the results include entropy effects and are thus a true measure of the affinity of the molecule to the surface.

## Methods

### Metadynamics Simulations

The four stable anhydrous forms of CBZ were included in the investigation, built from structures with the CSD identifiers CBMZPN11 (Form I), CBMZPN03 (Form II), CBMZPN10 (Form III), and CBMZPN12 (Form IV), as well as the dihydrate form, using a CSD structure reported as FEFNOT02, a monoclinic unit cell.^46^ Force field parameters for CBZ were taken from the generalized Amber force field (GAFF)^47^ with modified charges from quantum mechanics, and water was modeled by the TIP3P model.^48^ This combination was found to have the best performance in describing CBZ in both a solvent when evaluating the partition coefficient between water and octanol log*P* (cf. Figure S3) and in the bulk when looking at the density of the anhydrous crystal forms (cf. Figure S4). More details on the testing of the force fields are given in the Supporting Information. The stability of the solid crystals in the simulation was confirmed by running fully anisotropic NPT and observing the change in box vectors and angles (cf. Table S1). We then used this force field in all of the subsequent simulations.

The simulated systems consisted of 2D periodic slabs 3–4 nm thick containing 320–432 molecules of CBZ, 3.5–4.5 nm layer of water on top of the slab, and one additional molecule of CBZ placed in the water (hereafter referred to as “solute”). The surfaces were cut following the unit cell of the respective form by comparing them with how they would be cut in the VESTA software. For the 011 and 102, the cuts were made manually by cutting along the [011] and [102] directions, respectively, and excluding all molecules whose center of mass lies outside of the cut volume. This, of course, does not take into account all possible surface structures in a given direction, but a full exploration would increase the number of simulations to an amount that is beyond the scope of this study. Instead, the different surfaces serve as a tool to explore how the exposure of different parts of the molecule to the solvent affects the crystal growth strength. After a quick minimization of the constructed slab systems using the steepest descent algorithm, an equilibration in the NVT ensemble was run for 500 ps using the Berendsen thermostat^49^ at 300 K with a coupling constant of τ_*T*_ = 0.1 ps. The water in each box was then further equilibrated in an NPT simulation for 1 ns (velocity-rescale thermostat,^50^ Berendsen barostat^49^), applying a pressure of 1 bar in *z*-direction and 0 bar in all other directions, with a coupling constant of τ_*p*_ = 2 ps. Both the solute and the molecules in the slab were position restrained with a force constant of 1000 kJ mol^–1^ nm^–2^ during equilibration since previous unrestrained simulations had shown to introduce some edges and surface disorder to most surfaces (cf. Figures S9–S17) and the solute can only be initialized within a certain range due to the introduction of a wall (see below).

The metadynamics simulations were performed in an NVT ensemble using the same thermostat and parameters as the NPT run and a total simulation time of 1 or 1.5 μs, depending on convergence, starting from preliminarly equilibrated configurations. Now, the additional CBZ molecule acts as a solute that is free to move, while the others remain restrained in their slab positions with the same force constant as before to form the solid. All simulations were run in Gromacs 2021^51^ with a PLUMED 2.7^52^ patch and used the leapfrog integrator, with a time step of 2 fs. Nonbonded interactions were modeled with a cutoff of 1.4 nm and the particle-mesh Ewald method was used for long-range electrostatic interactions.^53^ All bonds containing hydrogen atoms were constrained using the LINCS algorithm.^54^

The CV was defined to be the surface separation distance (SSD) of the solute to the surface, which was computed as the difference between the *z* coordinates (perpendicular to the slab surface) of the center of mass of the solute and the center of mass of the slab, shifted by half the slab depth. The crystal faces for the slab surfaces were chosen to be the ones where the molecules on the surface expose the highest amount of their aromatic rings, i.e., where the molecules lie more or less “flat” on the surface. For Form I and the dihydrate, this coincides with the experimentally reported direction of crystal growth in the needle-shaped particles of these forms, their 100 and 001 surfaces, respectively^55,56^ (cf. Figures S5a and S7). Form II shows the same stacking as the relevant surface in Form I on its 001 face, which also coincides with the molecules lying “flat”, so it was the first choice for this form (cf. Figure S5b). For Form III, four more surfaces are sampled in addition to the originally chosen 010 surface (cf. Figure S6a) and the 100, 001, 011, and 102 surfaces (cf. Figure S8) due to the reported prismatic morphology of the crystals. For Form IV, only one surface was chosen, the 010 face (Figure S6b).

In the metadynamics simulations, Gaussians were deposited every 200 steps with a width of σ = 0.05 nm and a height of 0.01 kJ mol^–1^. The bias was stored on a grid with 1000 bins that was written to a file every 20 ns. The motion of solute toward the upper boundary is limited by a wall potential *k*_*i*_((*s* – *a*_*i*_)/*s*_*i*_)^*e*_*i*_^ for *s* > *a*_*i*_ that has parameters at *a*_*i*_ = 1.5 nm, with an energy constant *k*_*i*_ = 400,000 kJ mol^–1^, the exponent *e*_*i*_ = 4 and a rescaling factor *s*_*i*_ = 1 nm (offset is zero), while diffusion into the slab is restricted by a lower boundary with the same parameters, but at *a*_*i*_ = −0.5 nm. Form I was run with a slightly modified CV due to the small tilt of the 100 surface in the simulation box, which was necessary to make the box 3D periodic with the unit cell being triclinic. Instead of the SSD as described above, the minimum distance of the center of mass of the solute to any atom on the surface (defined as all residues in the first layer of the slab) was used as the CV.

After convergence of the metadynamics simulations (provided that the whole range of the CV was sampled repeatedly), the potential of mean force (PMF) was evaluated by integration of average force at the value of the CV or rather, in a small bin around given values of the CV.

### Quantifying Crystal Growth

The PMFs for the CV as defined in the previous section sample random adsorption of the solute molecule on the surface since the solute can approach and attach to the surface in any orientation. Conclusions about the crystal growth of the specific polymorphic forms are then reached by postprocessing of the trajectory, which allows us to distinguish states in the trajectory that attach in alignment with the underlying crystal structure and those which do not. Here, we introduce a special procedure to take into account only “correctly” aligned states and “rescale” the PMF so that it only shows the contribution of the aligned states and thus can be used for analysis of thermodynamic data for the crystal growth process.

In order to distinguish between orientations close to the surface and orientations far from it in the rescaled PMF, the data from the trajectory was divided into two sets. The first, hereafter referred to as bound states, comprised all states with an SSD inside the PMF minimum, i.e., the SSD range for which the original PMF of adsorption was below zero (more exactly below −0.9 kJ mol^–1^, to avoid including fluctuations in the bulk region). The second set, here referred to as unbound states, included the remaining states. Calculations for both sets were done separately as described below. All calculations for the selection of crystal growth states, both in the bound and in the unbound states, were carried out using in-house scripts that utilized the MDTraj library.^57^

### Determination of the PMF Compatible with the Crystal Structure

As mentioned before, metadynamics simulations using the SSD as described above provide the PMF and adsorption free energy of a solute at a surface; however, not all configurations of the solute near the surface are aligned with the crystal structure and represent crystal growth states. To quantify whether a certain adsorbed orientation of the solute molecule at the surface during the simulation corresponds to crystal growth, a set of criteria was applied to the bound states data set.

**Figure 2:**
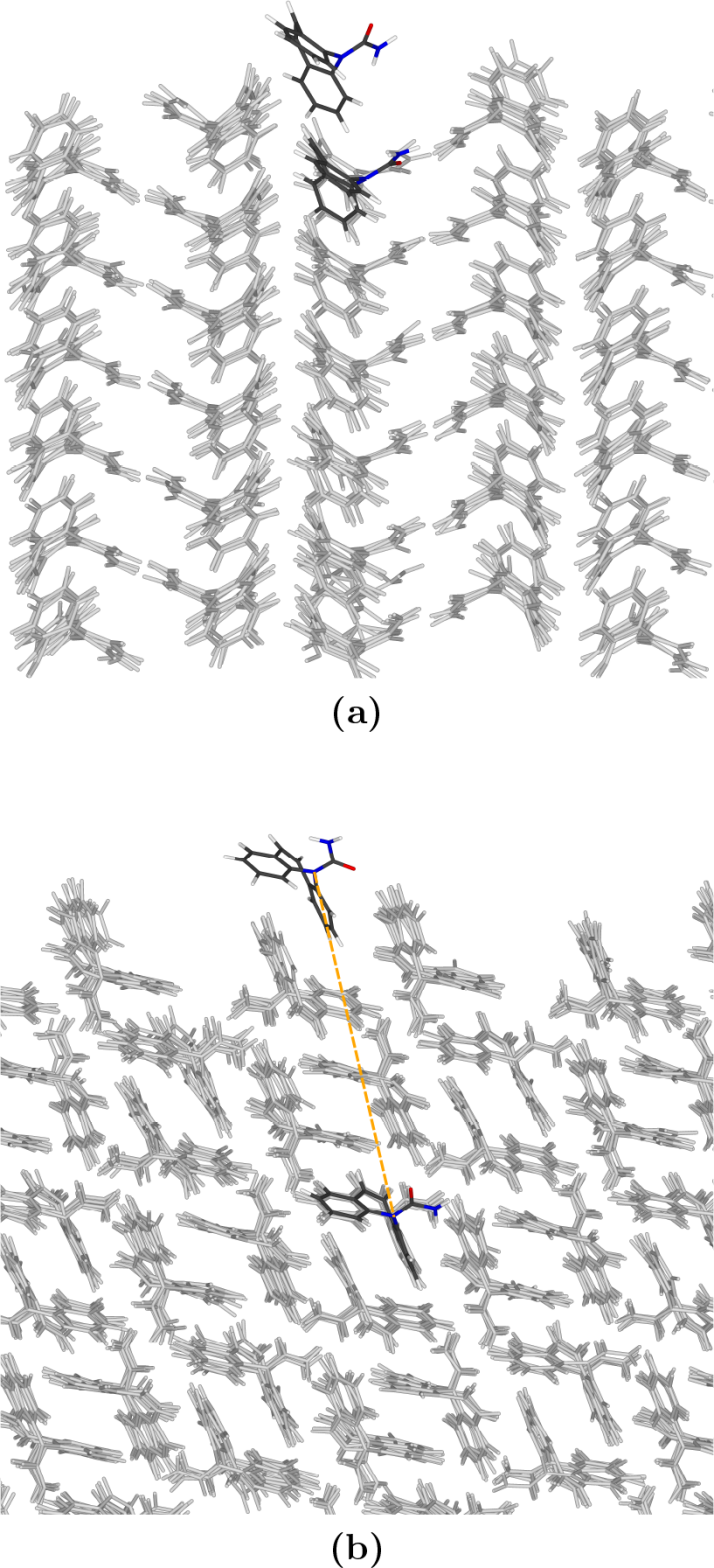
Different ways of stacking: (a) Molecules stack on top of each other, and the periodically repeating copy for the solute is in the first layer (here for the dihydrate). (b) Stacking in different orientations, the periodically repeating copy for the solute is further down in the slab (here for the 011 surface of Form III). (b) Also shows the projection of the solute along the *z* axis, which is used to find the nearest molecule in the periodically repeating layer. It should be noted that the amide group is not bound in the solute, so it can be rotated with respect to the slab molecule shown here.

First, we need to define the layer of the slab that would be the periodically repeating copy of a layer of new molecules at the surface. This could be the first layer, if the molecules simply stack on top of each other, or it could be further down in the slab (cf. Figure 2). Second, we select a molecule from the lower layer of the crystal cell such that the projection of the solute along the *z* axis to this plane is closest to this molecule, as illustrated in Figure 2b. It is supposed that the solute molecule adsorbed at the surface in the same orientation as the molecule chosen from the crystal slab in this way would be aligned with the crystal structure of the slab and can be considered as a crystal growth state.

**Figure 3:**
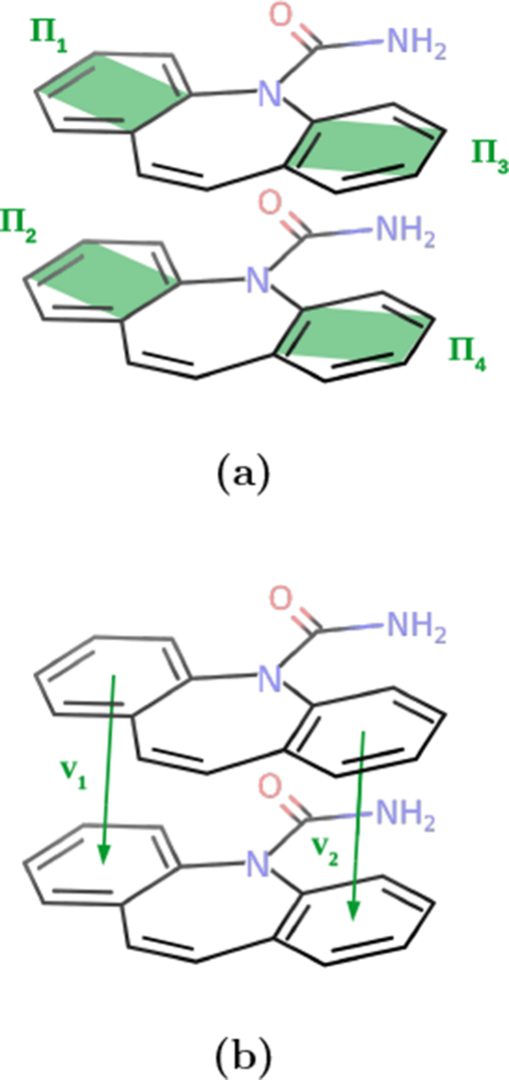
Aromatic ring planes (a) and center-of-mass vectors (b) for the rings in the solute and the nearest molecule in the slab. In each figure, the solute molecule is shown at the top, and the nearest molecule in the slab is shown at the bottom. Note that the molecules are shown as flat here for clarity, but the actual CBZ molecule will be bent in the seven-membered ring (cf. Figure 2). In the following text, the rings that constitute Π_1_ and Π_2_ will be referred to as ring 1 in the solute and slab, respectively, and the rings constituting Π_3_ and Π_4_ will be referred to as ring 3 in solute and slab, respectively. Ring 2 is defined as the seven-membered ring, but this ring is not included in the alignment tests.

Once the chosen molecule was determined, the angle of the planes of the six-membered rings was calculated. The planes are defined, as shown in Figure 3a, marked as Π_1_ to Π_4_. It should be noted that the indexing here does not refer to a fixed selection; rather, they are assigned on the fly based on the distances of the center of mass of the rings to each other. This is because the symmetry and orientational flexibility of the molecule make the two rings indistinguishable. Instead, two vectors connecting the center of mass of all rings are computed, shown in an example as ***v***_1_ and ***v***_2_ in Figure 3b, from which the center-of-mass distances of all combinations of six-membered rings in the solute and in the slab molecule were calculated. The corresponding rings are then selected by picking the combination of rings that minimizes the sum of the lengths of *v*_1_ and *v*_2_ to ensure that we only calculate angles between rings that are at similar distances. The distribution over the angles was evaluated and only states with angles less than 30° off the perfect alignment were selected. Since the ring system of the molecule can flip around during the simulation, this meant including angles between 0–30° and 150–180° in practice. For this selection, the center-of-mass distance of each six-membered ring in the solute to its equivalent in the slab molecule was calculated, i.e., the distance of the ring constituting Π_1_ to the one of Π_2_ and analogously of Π_3_ to Π_4_. The probability distribution over these distances was then calculated. It should be noted here that these are not the canonical (unbiased) angle/distance distribution but those obtained from the MetaD trajectory. To ensure the selection of all states that showed probabilities at least 5–7% of the maximum probability in the distribution, an ellipse was fitted to the maximum of each distribution, and the states in the ellipse were selected for rescaling.

A similar procedure was used to select states in correct alignment in the unbound set to get the distribution of states that were aligned with the surface but were further from it. As before, the alignment was calculated to the layer of the slab that showed the periodically repeating units of a new layer on the surface. This time, instead of calculating the angles and center-of-mass distances to the nearest molecule on the surface, they were calculated to all molecules in the surface layer of the slab (note that for some polymorphic forms, the surface contains molecules in different orientations). The same angle and distance criteria as in the bound set were applied to select the states for rescaling of the PMF.

The results of the alignment tests were then used to calculate the distribution of crystal growth states over the SSD with a fixed number of 100 bins. The PMF of the crystal growth attachment was calculated according to the algorithm described below. At the start, the full PMF of adsorption is defined as
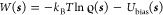
1where ***s*** denotes the CV and ϱ(***s***) is the probability distribution over the CV obtained in a converged metadynamics simulation with established bias *U*_bias_ (***s***). The rescaled (crystal growth) PMF is defined as
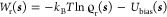
2where ϱ_r_ (***s***) is now the probability distribution of crystal growth states (i.e., aligned with the crystal structure) over the CV and normalized to the total number of states in the trajectory. Since ϱ (***s***) in a converged metadynamics simulation should be uniform over the CV, this term can be assumed to be constant and therefore be excluded. The PMF of crystal growth is then given by
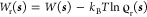
3

Since ϱ_r_ (***s***) includes only the aligned states but is normalized in the same way as ϱ(***s***), the rescaled PMF lies above the original PMF of adsorption. At large distances (in the bulk region where there is no effect of the surface), the rescaled PMF reaches a nearly constant value. This corresponds to the entropy contribution upon bringing the molecule from random orientation into the “correct” orientation at which the molecule is aligned with the crystal. Note that this also includes rotational entropy, i.e., if the molecule is in the same orientation as a molecule on the surface but they are rotated, for example, 90° to each other, the state is not counted as aligned, only if both rings are within 30° of each other is the state selected.

In fact, the molecules that were bound in aligned orientation and which are described by the crystal growth PMF adopt a random orientation if they detach and migrate to the bulk region, so they need to be described by the full PMF there. It is however difficult to select the orientational states that describe the attachment/detachment process in the region between the minimum of the rescaled PMF and the plateau in solution. This region is instead represented by a logarithmic function that interpolates between bound aligned states and free states far from the surface. It should be noted that the PMF in this region is not considered in any quantitative evaluation and the plot is purely for aesthetic reasons. Error estimates for the rescaled PMFs were obtained by block averaging with a block size of 2 ps (1000 frames) for the aligned states in the PMF minimum and 1000 to 3000 frames for the aligned states in the solution.

Conventionally the free energy change is computed from the PMF according to
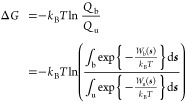
4where the subscript **u** denotes the unbound and **b** the bound states and integration is carried out over values of *s* corresponding to the bound and unbound region, respectively. Since we are interested in adsorption free energy of only aligned (crystal growth) states, we use the rescaled PMF *W*_*r*_(***s***) in the range of *s*, where the rescaled PMF is below zero (see Section 6.2 of the Supporting Information) for the description of the bound states. The unbound state is only considered to be at large distances where there is no effect of the surface and PMF can be taken as zero. We set therefore *W*_u_ (***s***) = 0 and obtain
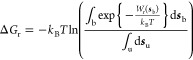
5

The integral is approximated as a sum over bins, so that
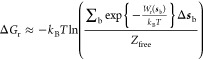
6where  is calculated using the trapezoidal rule from the area where the PMF curve is below zero. The thickness of the “free” area *Z*_free_ is generally defined by the bulk solute concentration. The “standard” adsorption free energy corresponds to the bulk concentration equal to 1 M. Here, we defined the thickness of the “free” volume as equal to the thickness of the bound layer. This corresponds to definition of adsorption free energy as “excess” free energy.

Since the entropy contribution is not dependent on the SSD at distances sufficiently far away from the surface, the rescaled PMF at those distances will fluctuate only around a certain value. The exact entropy contribution was then calculated by averaging over this long-distance part of the rescaled PMF:
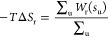
7

## Results and Discussion

**Figure 4:**
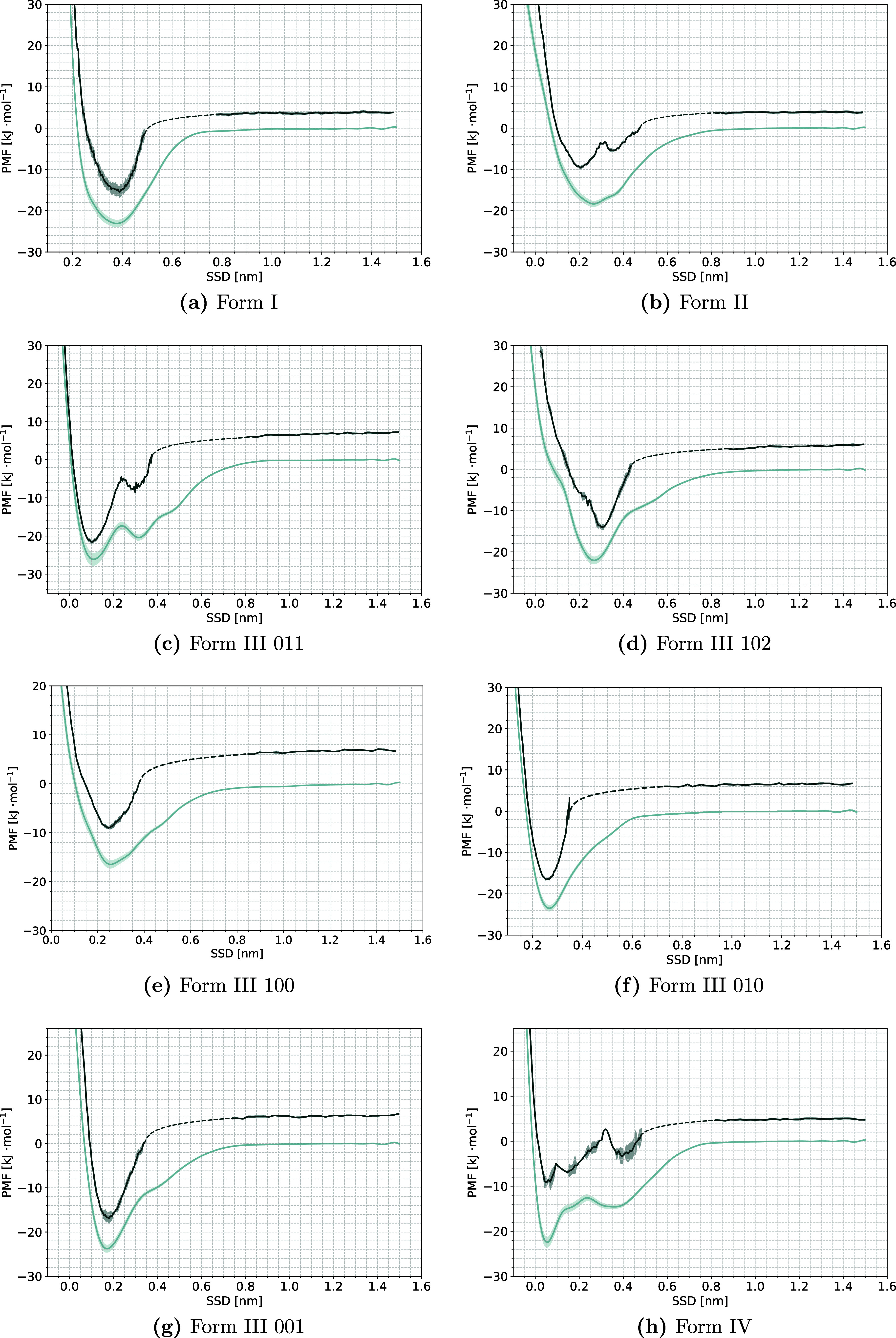
(a–h) Full PMF (light blue) and rescaled PMF for crystal growth states (dark blue) in all anhydrous forms. Shaded areas indicate error estimates in both curves.

**Figure 5:**
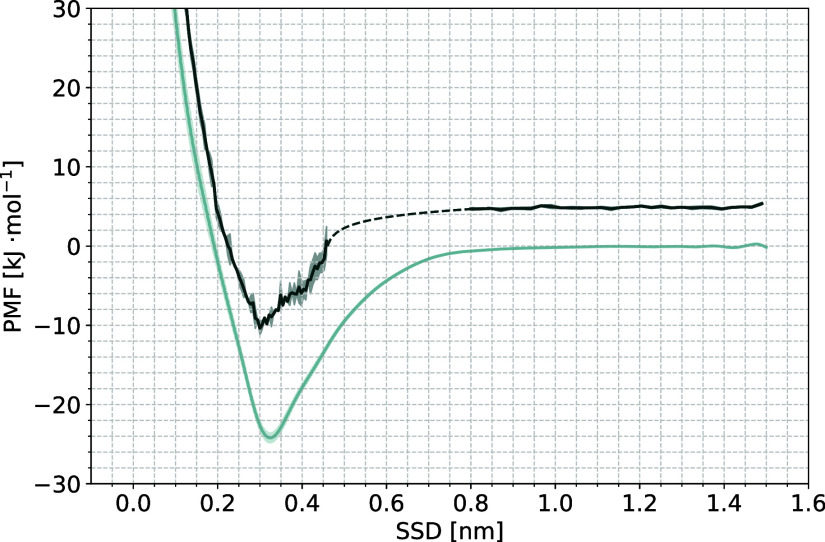
Full PMF (light blue) and rescaled PMF for crystal growth states (dark blue) in the dihydrate. Shaded areas indicate error estimates in both curves.

All metadynamics simulations showed good convergence (see Figures S18 and S19 of the Supporting Information). Comparing the volume covered by the solute over the course of the simulation to the available volume in solution in a procedure analogous to the one in Bertazzo et al.^58^ also showed that the full volume of the aqueous region was sampled (cf. Figures S21–S29 and Table S2 in the Supporting Information), so the correct entropy contribution in solution can be calculated from the simulation without correction. The full PMF and rescaled curves can be seen in Figures 4 and 5 for the dihydrate and anhydrous forms, respectively.

The free energy change upon adsorption is below −10 kJ mol^–1^ in all forms, meaning all show a minimum at the surface. Except for Form I and the dihydrate, all of the full PMF curves show secondary minima. While the secondary minimum is quite weak in most forms, it is very pronounced in the 011 surface of Form III, in Form IV, and to a lesser extent, in the 102 surface of Form III, indicating several clearly distinguishable sites of adsorption on the surfaces. The minimum of the rescaled PMF lines up with the minimum of the full PMF for most of the forms, but exceptions are seen in Form II, the 102 surface of Form III, and in the dihydrate. For all three, a drop in ϱ_*r*_ (***s***) can be seen close to the region of the full PMF minimum (see Figures S31, S33, and S38 in the Supporting Information), indicating that there is a competing mode of adsorption at this CV point, that does not correspond to crystal growth. It is the most apparent in Form II, where the rescaled PMF seems to show minima around the secondary minima of the full PMF, but not around the global minimum. Form II has characteristic empty channels in its crystal structure, which are thought to stabilize the structure when filled by solvents to increase packing efficiency.^59^ Some visual evidence was seen for the molecule adsorbing next to one of these channels away from the molecules of the surface, but this was not studied systematically.

**Figure 6:**
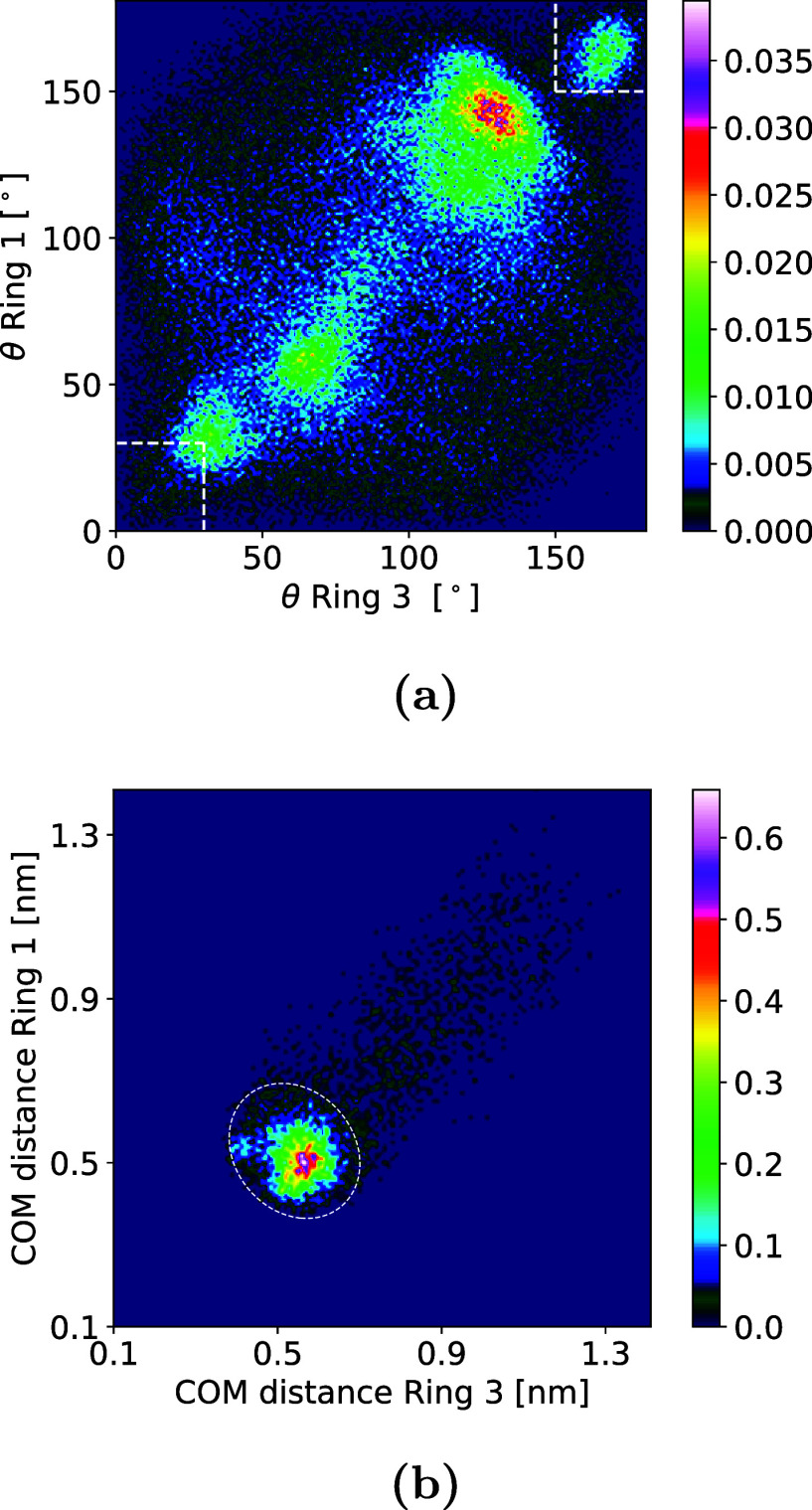
(a) Distribution of angles for states in PMF minimum for the dihydrate. Regions marked in white correspond to the correct alignment with the crystal structure. (b) Distribution of center-of-mass distances for states in the regions marked in white in (a) for the dihydrate (unit cell parameter perpendicular to the surface is *c* = 0.483 nm). Colorbars on the side show probability in %.

**Figure 7:**
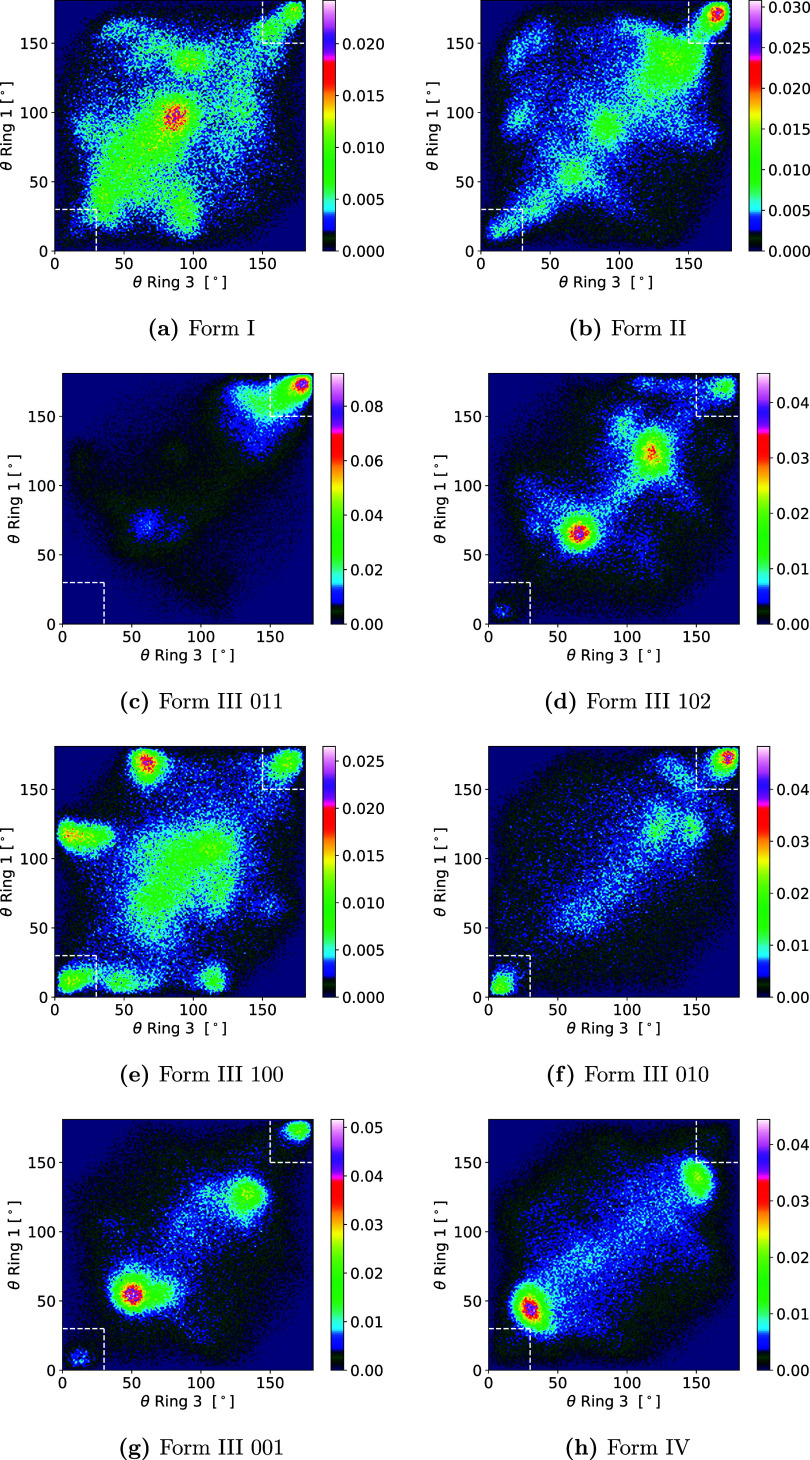
(a–h) Distribution of angles between six-membered rings for states in the PMF minimum in all forms. Colorbars on the side show probability in %. Regions marked in white correspond to correct alignment with the crystal structure.

In the selection of the crystal growth states and subsequent rescaling of the PMF, the distribution of angles between the six-membered rings and their center-of-mass distances were used as described in the Method section (Figure 6). The distribution of angles for the states in the bound data set can be seen in Figure 7 for all anhydrous forms and in Figure 6a for the dihydrate. A large amount of conformational flexibility for the solute close to the surface is seen for the 100 surface of Form III, for Forms I and II, and for the dihydrate, with multiple peaks and a large spread of values in the angle space. The 001 and 102 surfaces of Form III as well as Form IV show two to three major peaks and little spread, while the 010 and 011 surfaces of Form III show very little flexibility, with all major peaks in the regions marked in white (correct alignment of the rings with the underlying crystal structure).

**Figure 8:**
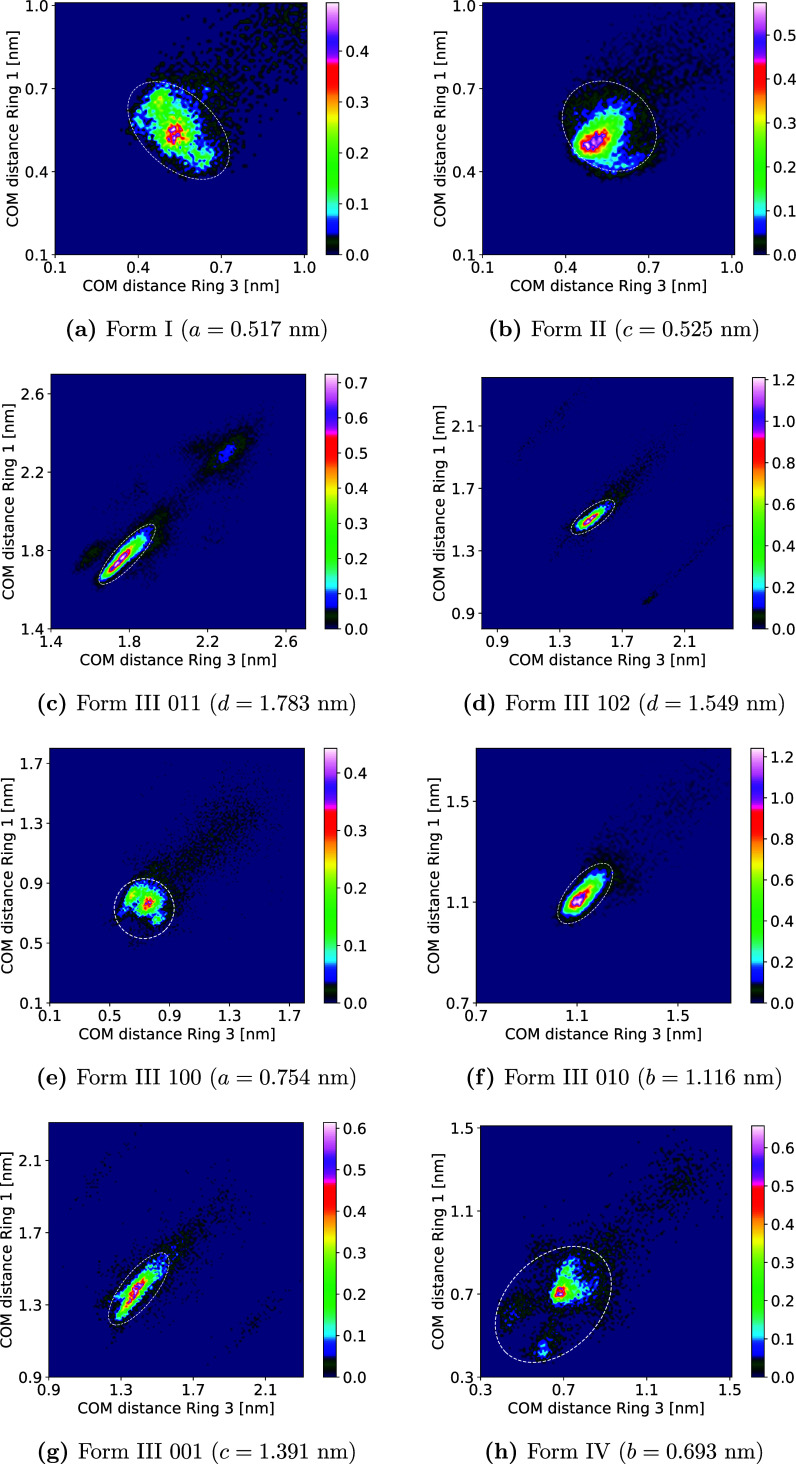
(a–h) Distribution of center-of-mass distances between six-membered rings for states in the regions marked in white boxes in Figure 7 in all forms. Colorbars on the side show probability in %, values in the subcaptions show the unit cell parameter in *z* direction (perpendicular to the surface).

The distribution of center-of-mass distances between the six-membered rings for all states in correct alignment in the angle distributions is shown in Figure 8 for all anhydrous forms and in Figure 6b for the dihydrate. All show pronounced maxima around the unit cell parameter for the direction perpendicular to the surface or the distance between repeating layers in the case of the 011 and 102 surfaces of Form III. Form IV shows two smaller maxima at closer distances than the unit cell parameter for its 010 surface. These were visually confirmed to be just alternate rotations of the molecule plane and were counted as aligned with the crystal structure (cf. Figure S41). The smaller maxima in the 011 surface of Form III however were excluded from the aligned state calculation, since they proved to be too far rotated (smaller maximum to the left of the primary maximum) or not the correct orientation (larger maximum around 2.3 nm in both rings), as seen in Figure S39. The latter proved true also for the “satellite” states in the 001 and 102 surfaces of Form III (cf. Figure S40). The amount of states that could be included as crystal growth states in this way was usually below 10% of all states in the bound data set, except for the 010 and 011 surfaces of Form III, where they represented around 13 and 16%, respectively (cf. Table S3). The lowest amount was found for Form IV with around 2.5%, which together with the separated peaks in Figure 8h points to lose attachment when aligned with the crystal structure. The solute is not bound strongly, so large fluctuations in the orientation occur and lead to many states being detached further and, therefore, not being selected. Interestingly, all polymorphs which show a stacked attachment (molecules stack on top of each other in the crystal structure; this is true for Forms I, and II, the chosen surface of Form IV and the dihydrate) all display a greater variability of distances when comparing ring 1 and ring 3. In contrast, the Form III surfaces, where multiple orientations in the direction perpendicular to the surfaces are seen, show narrow distance maxima, where the molecule can be attached at slightly different distances overall, but the distances of the two rings do not differ much. The only exception to this is the 100 surface, which seems to be more complex so that molecules can not attach as strongly as on the other surfaces.

**Figure 9:**
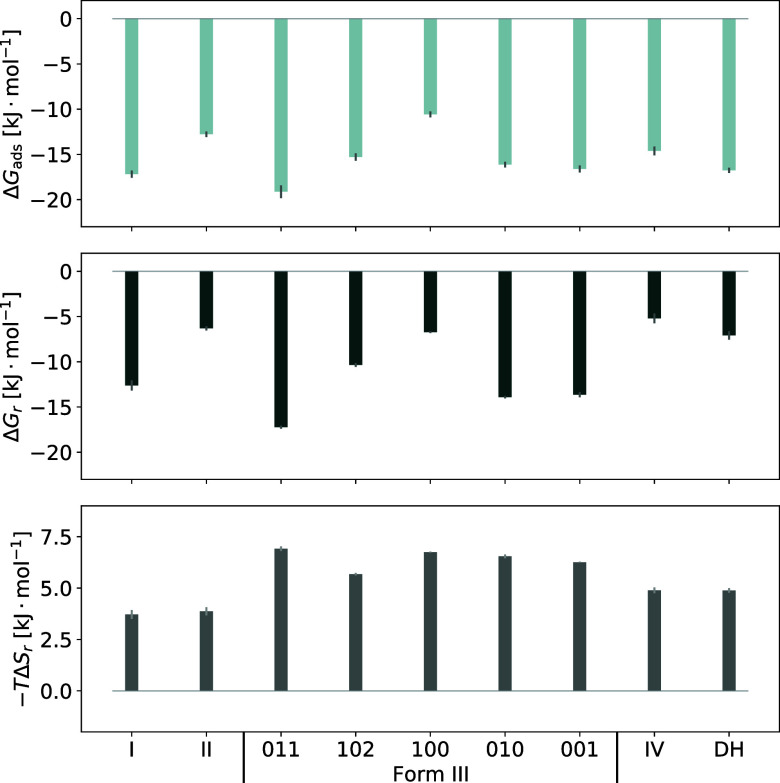
Graphical summary of Table 1 entries.

The adsorption free energies obtained from the full PMFs in all forms are listed in the first column of Table 1, together with the free energy changes obtained from the rescaled PMF. The distribution of aligned states in the PMF minimum and the area of the rescaled PMF that was integrated to obtain the free energy change Δ*G*_*r*_ can be seen in Figures S30–S38. The strength of adsorption decreases as I > DH > IV > II, with the average in Form III surfaces somewhere between the dihydrate and Form IV. The large difference in values between Forms I and II is somewhat surprising given their similarity, but this is probably due to the effect of the empty channels in Form II. Within Form III, the order of adsorption strength is 011 > 001 ≈ 010 > 102 > 100. The magnitude of the free energy change from the rescaled PMF is in the order I ≫ DH > II > IV, with the average of Form III being somewhere close to Form I. Within Form III, the free energy increases as 011 > 010 ≈ 001 > 102 > 100, analogous to the decrease in adsorption strength in the full PMF. The three surfaces that show the most favorable total free energies are the 011, 010, and 001 surfaces of Form III and the 100 surface of Form I, making them the most suitable for crystal growth. Others show higher free energy changes like Form II, the 102 and 100 surfaces of Form III and the 001 surface of the dihydrate. Form IV shows the smallest (least negative) free energy change, making it the thermodynamically least likely for crystal growth (cf. Figure 9). This means in turn that the rate of dissolution, which can be considered the inverse process of crystal growth, goes in the order IV > II > DH > I, with Form III being between DH and I on average. For the surfaces of Form III, the ranking is 100 > 102 > 001 ≈ 010 > 011.

**Table 1:** Free Energy of Adsorption, Free Energy Change in Rescaled PMF, and Entropy Contribution of Crystal Growth Attachment in All Forms (All Values in kJ mol^–1^)

form	surface	Δ*G*_ads_	Δ*G*_r_	–*T*Δ*S*_r_
I	100	– 17.19 ± 0.40	– 12.62 ± 0.57	3.72 ± 0.22
II	001	– 12.78 ± 0.31	– 6.31 ± 0.11	3.87 ± 0.20
III	011	– 19.13 ± 0.71	– 17.23 ± 0.18	6.92 ± 0.11
	102	– 15.30 ± 0.41	– 10.35 ± 0.22	5.68 ± 0.06
	100	– 10.58 ± 0.33	– 6.73 ± 0.11	6.75 ± 0.03
	010	– 16.13 ± 0.32	– 13.91 ± 0.14	6.55 ± 0.09
	001	– 16.62 ± 0.39	– 13.64 ± 0.29	6.26 ± 0.02
IV	010	– 14.62 ± 0.49	– 5.20 ± 0.56	4.89 ± 0.14
DH	001	– 16.77 ± 0.29	– 7.08 ± 0.48	4.89 ± 0.12

The entropy contributions match in Forms I and II and in Form IV and the dihydrate as well as for most surfaces of Form III (011 and 102 surfaces show slightly higher/lower contributions). This gives insight into how many unique orientations each surface consists of. Form I and II, because of their more simple packing perpendicular to the surface, show a greater variety of orientations in the plane of the surface, which lowers the entropy contribution needed to move the solute molecule into alignment with one of them. Form IV and the dihydrate show slightly fewer orientations, in the former due to the more complicated packing perpendicular to the surface and in the latter due to a very regular pattern of packing in the plane that increases entropy by limiting the number of orientations. Form III only shows two distinct orientations on all of its surfaces and therefore the highest entropy; however, these are very similar in the case of the 011 surface, leading to a slightly higher entropy contribution. The remaining surfaces all show similar entropy contributions, with the exception of the 102 surface. Looking closely at Figure 4d, the rescaled PMF in the bulk region seems to reach another plateau at around 1.4 nm, so it is likely that this surface would also reach an entropy contribution of about 6 kJ mol^–1^, but the effect of the surface on the solute is more long-ranged than for other surfaces.

Since the criteria of crystal growth are based only on the alignment of the six-membered rings, there are three distinct ways the molecule can be oriented at the surface: (i) full alignment of all atoms in the molecule of the slab to the solute, (ii) full alignment of the ring system, but the amide group in the solute is rotated 180° with respect to the amide group in the molecule at the surface, and (iii) the ring system (symmetric) in the solute is rotated 180° with respect to the ring system in the molecule in the slab (cf. Figure S42). All of these are counted, as all of them would contribute to crystal growth states, albeit forming defects. The amount of contribution of the three orientations was investigated using two intramolecular vectors (see Figure S43 of the Supporting Information) and revealed that most forms prefer the full alignment or a mix of full alignment and the rotated amide (cf. Figures S44–S52). Since there is enough rotational freedom in the amide even at the surface, orientation (ii) would likely not introduce any defects. However, for Form II and for the 102 and 010 surfaces of Form III (to a much lesser extent also the 011 and 100 surfaces of Form III), orientation (iii) was dominating, indicating that on these defects growth might be favored.

### Solubility and Dissolution

Kobayashi et al.^19^ found the bioavailability of Form III to be higher than those of the dihydrate and Form I when administering a 200 mg/body dose in dogs. Xu et al.^60^ found a higher bioavailability for Form I than Form III in rats, which they pose as a better predictor for the bioavailability in humans. Kipouros et al.^61^ measured the dissolution rate of Forms III and IV (∼150 mg) in simulated gastric fluid (pH 1.2) and found the former to be higher. Kaneniwa et al.^62^ found the dissolution rate of Form II to be higher than for Form III in water, which was in turn higher than that of Form I. From the literature data, it is not possible to determine what the relationship is between all anhydrous forms and the dihydrate regarding their solubility, but hydrates are generally less soluble than their anhydrous counterparts,^63^ and at least Form III is known to be more soluble than the dihydrate.^64^

From our rescaled PMF data, Form IV and the dihydrate are predicted to have the highest affinity for dissolution, which is not seen in practice. It should, however, be noted that some of the experimental results were obtained in the presence of other hydrophobic molecules and sometimes varying pH, so they should not be directly comparable. Moreover, the comparison of dissolution, a process, and solubility, a defined equilibrated concentration, is not straightforward. Lastly, this study is considering the attachment of a single molecule to a specific, flat surface, which may not be directly proportional to the overall solubility, since real surfaces may have a larger surface area and edges, defects, and the general morphology of the crystal will play a role. Moreover, the limiting step will be dissolution from the largest surface of the particle, which is synonymous with the slowest growing face of the crystal and, therefore, not considered in our study for most of the forms. A more accurate approach to solubility can be derived from the dissolution of the solute from a kink site at the slowest growing surface, as proposed by Bjelobrk et al.^32^ for the solubility of urea and naphthalene in different solvents. However, this is beyond the scope of this study since the main goal was to investigate the aligned attachment in the crystal growth process and not the solubility.

On a general note, Gadelmeier et al.^38^ also reported the molecular mechanics parameters of a modified GAFF failing to describe Form IV well in particular, so this form might be dominated by interactions that are not well described by classical force fields.

### Crystal Growth

Overall, it was found that while crystal growth is possible in all forms, some of them are thermodynamically favored. Moreover, even when the molecule is at the surface, there can be some orientations that are very similar to the crystal structure but slightly different, such as the solute being rotated by 180° with respect to the positions in the crystal structure. The amount of states in this orientation, which would lead to defects or similar disruptions, is highest on the 010, 102, and 011 surfaces of Form III and in Form II. Laine et al.^16^ reported that the dihydrate can grow on the surface of Form III and Murphy et al.^64^ later showed that defects and amorphous regions on the surface can promote this growth. The rotated orientation of attaching molecules on certain surfaces of Form III in water could be the starting point of such defects that lead to the formation of the dihydrate since it is found in more than half of the surfaces investigated here, among them two of the most likely ones for crystal growth.

From the rescaled PMF and the free energies of crystal growth, it seems that Forms IV, II and the dihydrate are not preferred crystallization products in water. However, for both Form IV and the dihydrate, evidence has been found that they proceed through amorphous stages or liquid prenucleation clusters and not through a classical growth mechanism.^65,66^ Studies on Form II crystallization in the presence of additives have shown that crystallization in this form is promoted by the stabilization of molecules along the empty channels.^67,68^ It is likely that water as a solvent is inadequate to stabilize such channels formed by the hydrophobic parts of the molecule, which would explain the low propensity for crystal growth in our results. In fact, most of the additives that seem to promote growth in Form II are characterized by long hydrocarbon chains such as sodium stearate. The strong crystal growth of Form I in this study is not observed in the experiment either, but this is most likely related to the low thermodynamic stability of this polymorph,^10^ which leads to it not being crystallized in water. Form III, on the other hand, has been observed in the crystallization from an aqueous solution, albeit in small fractions,^66^ showing some influence of the thermodynamics on the final products.

As with the solubility, the importance of edges, kinks, surface area, etc., on the thermodynamics of crystal growth can not be neglected, which are not captured here due to the simplification of surfaces that was done in order to set up the simulations. Another factor that is not taken into account here is concentration since this has been shown to shift the kinetics in favor of different polymorphs.^18^ However, this idealized system can still give some insight into the differences in the energetics and the dominating interactions in the different polymorphs and crystal faces. In fact, looking at what unites the surfaces that are thermodynamically favored and show the strongest crystal growth in this study, it seems that the hydrophobicity of the surface plays a major role, which has previously been reported to be the dominant interaction in organic crystals containing aromatic and hydrogen-bonding moieties.^69^ The most hydrophobic of all is the 011 surface of Form III since it has no hydrogen bonds exposed and the only interaction that can occur is via the nonpolar ring system. A caveat here is that this exposure of the hydrophobic parts is unlikely to occur in this way in water because of the unfavorable interaction. In fact, the 011 surface was the only one where dissolution in the unbiased simulations went beyond the first layer on the surface (see Figure S11), showing that this surface is unstable in water and not the primary site for crystal growth. Forms I and II both show a balance of hydrogen bonding and aromatic interactions on their 100 and 001 surfaces, respectively,^10^ but again the surface area of the aromatic rings is large. However, Form II is characterized by large channels in the *z* direction, which introduce a competing mode of adsorption, as discussed previously. Furthermore, the channels might actually destabilize adsorption states altogether, since the overall adsorption free energy in Form II is among the lowest in all forms. The 010 and 001 surfaces of Form III show similar values to Form I and they are equally characterized by a balance of hydrophobic and hydrophilic interactions at the surface. The same is true for the 102 surface, but the propensity for crystal growth is decreased by a competing adsorption mode. The 100 surface is the most particular in Form III, as it shows the lowest overall adsorption free energy of all investigated surfaces. It has been reported before that the 100 surface is the slowest growing surface of Form III when crystallized from methanol,^70^ so it is expected that this would also be the case in water, as both are polar protic solvents. Interestingly, the attachment energy of Form III in a vacuum suggests that the 100 surface should be a preferred site, since it shows a lower attachment energy than the 011 surface for example,^71^ which could be another sign that our model is able to describe their affinities in solution more accurately. In principle, the surface area of the ring system on the 001 surface of the dihydrate is also quite large, but it has very few sites of attachment due to the space occupied by the crystal water, so the unfavorable thermodynamics might have a statistical cause. Lastly, the 010 surface of Form IV seems to mostly suffer from weak interactions when attached to the surface, as discussed before. Though the crystallization might be more favorable along a different surface, the experimental evidence suggests that Form IV does not crystallize through a classical pathway anyway.

## Conclusions

Five carbamazepine polymorphs were investigated by using the adsorption of a molecule to a flat surface as a model. Subsequent postprocessing of the trajectories and rescaling of the obtained PMF allowed for the separation of thermodynamic contributions into those of randomly adsorbed states and states corresponding to crystal growth. The main crystallization product, the dihydrate, was not found to be among the thermodynamically most likely ones, which could confirm that this form follows a nonclassical crystallization process. Forms IV and II were also found to be thermodynamically less favorable, which could either confirm the nonclassical growth from amorphous carbamazepine in Form IV, or be further evidence that this form is not modeled well by molecular mechanics, as some influences on the process are not captured by the model (e.g., polarizability). Based on previous literature findings, Form II is hypothesized to only crystallize in the presence of some hydrocarbon moiety either in the solvent or in additives that is able to stabilize the growth around empty channels in the crystal structure of this form. The most favorable energetics were seen for Forms I and III, but since the former is only crystallized at high temperatures, there is likely some kinetic barrier in the nucleation process. Much of the thermodynamics of a surface seems to be related to its hydrophobicity and the maximization of hydrophobic interactions with the solute, which is in line with previous results on organic molecules containing both hydrogen-bonding and aromatic groups.

Future studies will hopefully enable us to explore further dependencies on the choice of solvent, presence of additives, etc., of the crystallization of the CBZ polymorphs, which might be a jumping-off point for other organic molecules, exhibiting the same structural complexity or packing polymorphism. As this hinges on the development of a CV that will accurately distinguish the polymorphs from amorphous or solvated states and from each other, the focus should be on the investigation of all their structural and physical differences that could be used to effectively differentiate between them during the nucleation process.

## Supporting Information Available

Dimerization in water and stability of slabs in unrestrained simulations; testing of force fields using the water–octanol partition coefficient, the solid density, and the energy of formation; visualization of investigated surfaces; plots of convergence of force-integrated PMF to stored bias and sampling of the CV over the simulation time; sampling of the volume by the solute; statistics on frequency of alignment in the bound data set, distribution of found states over the CV, and visualization of the subset of the rescaled PMF used to integrate the free energy change; visualization of unusual alignments that lead to secondary maxima in the COM distance distributions of Forms IV, III 011, 102, and 001; alternate rotation state definitions, quantification, and distributions for all forms (PDF)
