# Supplementary material for: Exploring Carbamazepine Polymorph Crystal Growth in Water by Enhanced Sampling Simulations
Source: ACS Omega. 2024 Aug 16;9(34):36718–31. doi: 10.1021/acsomega.4c05458 (PMC11360048; doi:10.1021/acsomega.4c05458)
Supplement: Supplementary file 1 — ao4c05458_si_001.pdf [file ao4c05458_si_001.pdf]

Exploring carbamazepine polymorph crystal growth in water by enhanced sampling simulations - Supporting Information

Radost Herboth* and Alexander P. Lyubartsev*

Department of Materials and Environmental Chemistry, Stockholm University, Svante Arrhenius väg 16C, 106 91 Stockholm, Sweden

E-mail: radost.herboth@mmk.su.se; alexander.lyubartsev@mmk.su.se

Dimerization in water

The fundamental unit of all anhydrous polymorphs of CBZ is the hydrogen-bonded dimer, in which the amide groups of both monomers are hydrogen-bonded in a ring motif (cf. Figure S1 (a)), but other patterns are seen in some of the forms. In solution, it can form dimers of solutes in different ways depending on the solvent, both through interactions of the amide group by hydrogen bonding and the weak interactions of the aromatic rings.

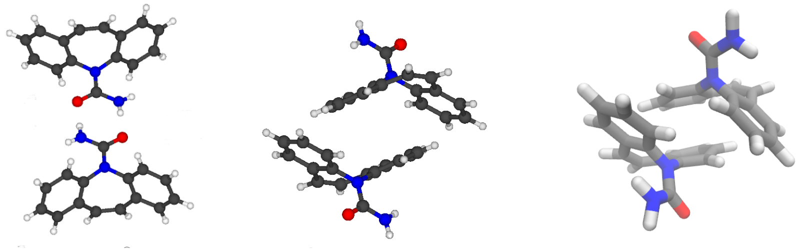

(a) **(b)** **(c)**

Figure S1: Hydrogen-bonded dimer/fundamental unit (a), π-stacked dimer found in Forms III and IV (b) and dimer motif in the supersaturated solution (c).

As shown by Hunter *et al.*[1] in solutions of deuterated methanol - a polar solvent - CBZ dimerizes in a way that the contact of the non-polar rings with the water is minimized. In deuterated methyl chloride instead, the dimer mentioned in the previous paragraph appears, since the formation of hydrogen bonds between the CBZ molecule is now a more favorable interaction. To determine whether the same occurs under the conditions used in our systems, a simulation in water at a supersaturated concentration was run.

This simulation did not show the dimer as in Figure S1 (a), there were hydrogen bonded configurations, but none of them exhibited the ring motif, there were only single hydrogen bonds. As seen in Figure S2, the center-of-mass distance is also much lower than the average distance of the amide groups to each other, which implies that if a dimer is formed, it is by the interaction of the non-polar ring system, which is expected since water is a polar solvent, similar to methanol, and disrupts the formation of hydrogen bonds between monomers. Indeed, upon visual inspection a π-stacked dimer is seen that resembles the pattern in Form

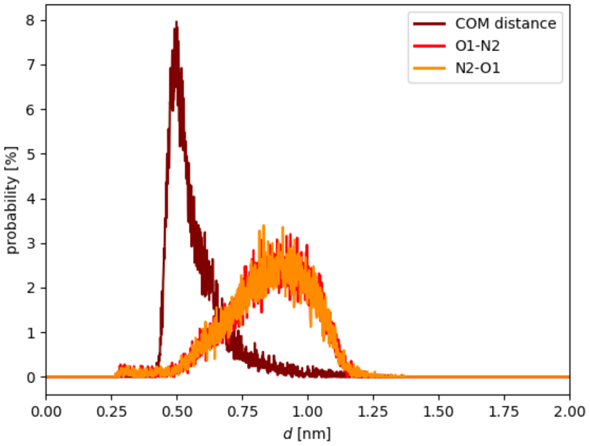

Figure S2: Distribution of distances in the supersaturated solution of CBZ in water. O1 and N2 are the names of the heavy atoms involved in hydrogen bonding.

III and IV (see Figure S1 (b) and (c)).

Testing of forcefields

Two conventional force fields, OPLS-AA (Optimized Potentials for Liquid Simulations)[2] and the generalized Amber force field (GAFF)[3] were tested using different charge calculation schemes (AM1BCC and RESP) before choosing the most adequate one. The molecules in both the solution and the solid form were investigated and the performance of the force field was assessed by computing density, the partition coefficient logP, and the energy of formation. The RESP charges were obtained from the electrostatic potential calculated in *Gaussian 16*[4] with a HF/6-31G* basis set for a molecule in a dielectric continuum representing the water environment ($\epsilon = 78.3553$), and exported to Gromacs input with the *ACPYPE* utility.[5]

Partition coefficient logP

The partition coefficient $\log P_{ow}$ for CBZ was calculated using

$$\log P_{ow} = \frac{(\Delta G_w - \Delta G_o)}{RT} \cdot \log e \qquad (1)$$

where ΔG_w is the solvation free energy in water, ΔG_o the one in octanol, R the universal gas constant (8.31446261815 J $\cdot$mol$^{-1} \cdot$ K^{-1}), T the temperature (300 K) and e Euler's number.[6]

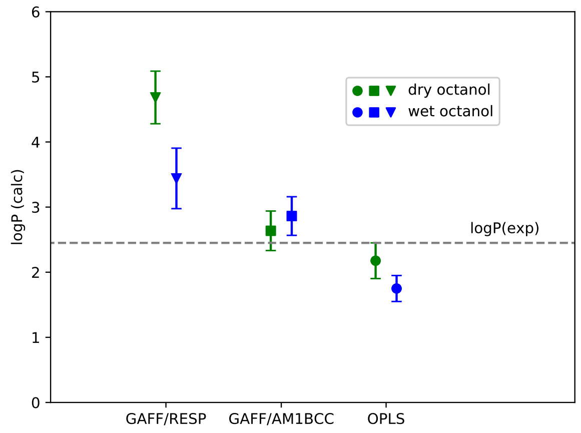

Figure S3: Matching of logP data from the simulation and experimental logP for CBZ (logP = 2.45). Calculated results were obtained both from pure octanol (dry octanol) and octanol with 27 mol% water (wet octanol).

Since the solubility of water in octanol is non-negligible,[7] the solvation free energy in octanol was calculated both for the pure compound ('dry octanol') and for octanol with 27 mol% water ('wet octanol'). Parameters and coordinate files for both dry and wet octanol were taken from the MDPOW package (`https://github.com/Becksteinlab/MDPOW`) for OPLS, which uses refined parameters for octanol developed by Zangi.[8] For GAFF, all topologies were generated using `antechamber`.[9] Results are plotted in Figure S3. For the GAFF/RESP calculations, the RESP charges of CBZ were calculated both in water as described before and in octanol using a dielectric continuum representing n-octanol ($\epsilon = 9.8629$) and used for the calculations in the respective solvents. The charges obtained from the octanol environment were used both for dry and wet octanol. Values show good agreement with experimental data for GAFF/AM1BCC and OPLS, slightly less so for GAFF/RESP. The latter is also much more sentitive to the choice of solvent, which makes sense considering the charge calculation. However, the calculated value for GAFF/RESP in wet octanol still

comes reasonably close to the experimental value.

Density in the solid form

The densities of the four anhydrous forms of CBZ were calculated from simulations in the crystal phase. Unit cells for all forms were obtained from the Cambridge Structural Database (CSD) and were then replicated in a modified Atomic Simulation Environment (ASE)[10] script, yielding simulation boxes that contained between 216 and 432 residues. For Form II, hydrogen atoms had to be added in *OLEX2*[11] using the command `hadd`.

Table S1: Change in box vectors and angles in % from NVT to anisotropic NPT in GAFF/RESP.

Form	a	b	c	α	β	γ
I	0.62	2.95	-0.36	-0.81	0.94	-2.17
II	-0.02	-0.01	-0.21	0.0	0.0	0.0
III	1.93	1.91	-3.10	0.0	3.77	0.0
IV	4.07	-3.07	5.00	0.0	3.45	0.0

Parameters in the force fields were the same as in the simulations of CBZ in water. Each form was first minimized using a steepest descent algorithm and equilibrated in a NVT ensemble for 100 ps using the velocity rescaling thermostat.[12] They were then run in a NPT ensemble with anisotropic pressure scaling for 1 ns, using the the same thermostat as before and the Berendsen barostat.[13] A change in box size was observed upon switching to anisotropic scaling, however, the fluctuations during the simulations were small ($<= 5\%$, see table S1 for GAFF/RESP), so the crystals are stable in simulation. The average density of each system in anisotropic pressure scaling was calculated, the results are plotted in Figure S4.

The general trend in all force fields is the same: Forms I and III show higher densities ($> 1.3\,\mathrm{g \cdot cm^{-3}}$), while both Form II and Form IV lie below or around 1.24 $\mathrm{g \cdot cm^{-3}}$. This makes sense looking at the crystal packing in I and III vs. in II and IV. Both of the latter show gaps in the unit cell, Form II in along c and Form IV along a. In contrast, Form I and

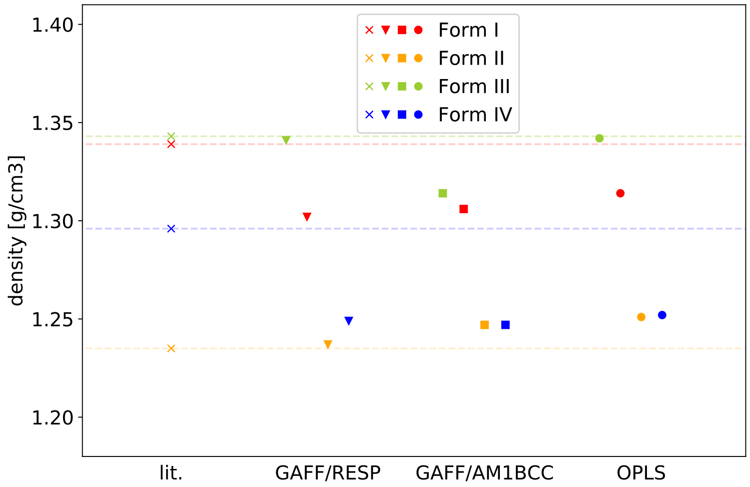

Figure S4: Density of the solid forms of CBZ as obtained from simulation as compared to density in the CIF file obtained from the CSD.

Form III show a more compact and efficient packing, which makes higher densities plausible. This trend is also seen in the colored 'x' markers, which are the densities as obtained from the CIF files of each respective form. The best agreement with the CIF numbers is seen for Form II and Form III, but overall, the densities are reproduced within reasonable limits and especially the GAFF/RESP force field performs well.

Energy of formation

A last test was to evaluate the energy of formation in GAFF/RESP and compare it to experimental data on the thermodynamic stability as given by Grzesiak et al. The energy of formation is calculated as the potential energy difference between the energy per molecule in the (minimized) solid and the energy of an isolated molecule in vacuum as

$$E_{\text{form}} = (E_{\text{tot,solid}}/N_{\text{mol}}) - E_{\text{isol,vac}}. \tag{2}$$

Grzesiak et al. report thermodynamic stabilities in the order III > I > IV > II.[14] The energies of formation calculated from the minimizations of the solids were $-123.14\ \text{kJ} \cdot \text{mol}^{-1}$ for Form I, $-120.57\ \text{kJ} \cdot \text{mol}^{-1}$ for Form II, $-125.58\ \text{kJ} \cdot \text{mol}^{-1}$ for Form III and $-114.3\ \text{kJ} \cdot \text{mol}^{-1}$

for Form IV, which puts them in the order III > I > II > IV. It seems therefore that GAFF/RESP reproduces the experimental order reasonably well.

Setup of slabs

The crystal structures of the relevant surfaces are shown in fig.s S5-S8. In all simulations, z is defined as the component that is perpendicular to the surface. In Form I this is the x component (100 surface), since it has been reported to be the main direction of crystal growth in Form I upon sublimation.[15] The 100 surface of Form I and the 001 surface of Form II show the same type of stacking of molecules and both forms show needle-like crystals, so it can be inferred that crystal growth in Form II will proceed along z, thus this direction was chosen. In Form III and IV the y component (010 surface) was originally chosen due to the orientation of the surface, since the molecules are mostly flat on these surfaces, but more were added for Form III due to the reported prismatic morphology of the crystals. The 001 direction was chosen for the dihydrate since it has been reported to be the main direction of crystal growth in this form.[16]

Slabs of the crystal forms were created using the same unit cells as before, replicating them in the modified ASE script and adding water on top of the slabs using the `gmx solvate` utility of Gromacs.[17] The 011 and 102 surfaces of Form III were cut manually and the resulting slab was rotated using `gmx editconf` to have z perpendicular to the surface. Since this rotation can introduce small misalignments in the crystal, they were then equilibrated for 1 ns in a NPT simulation, before adding water on top and equilibrating the full system.

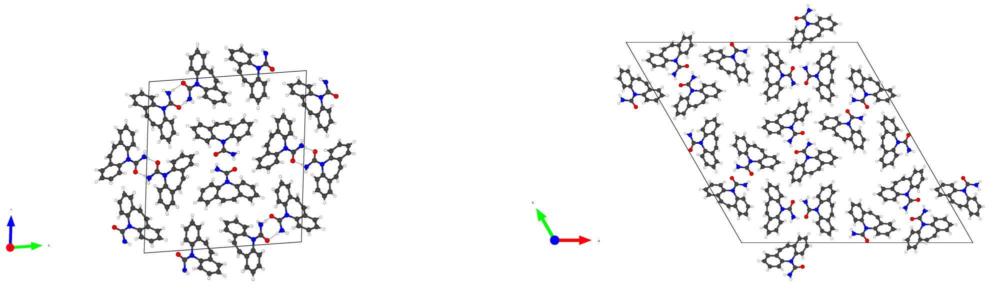

(a) Form I **(b)** Form II

Figure S5: Surfaces for Forms I and II of CBZ (top-down view).

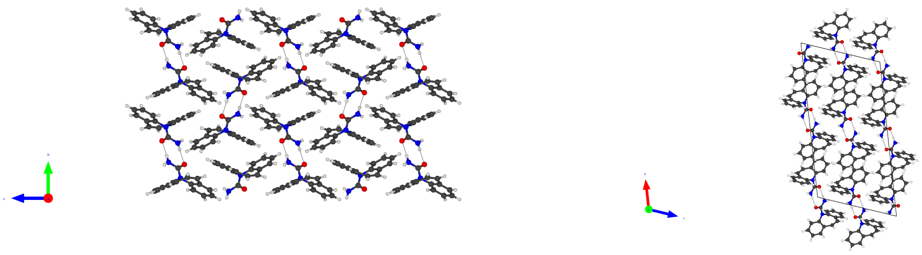

(a) Form III **(b)** Form IV

Figure S6: (010) surface for Forms III (side view) and IV (top-down view) of CBZ.

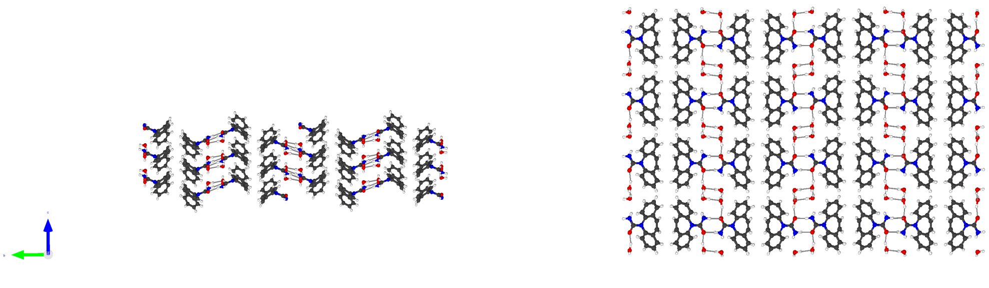

(a) side view **(b)** top-down view

Figure S7: 001 surface of the dihydrate.

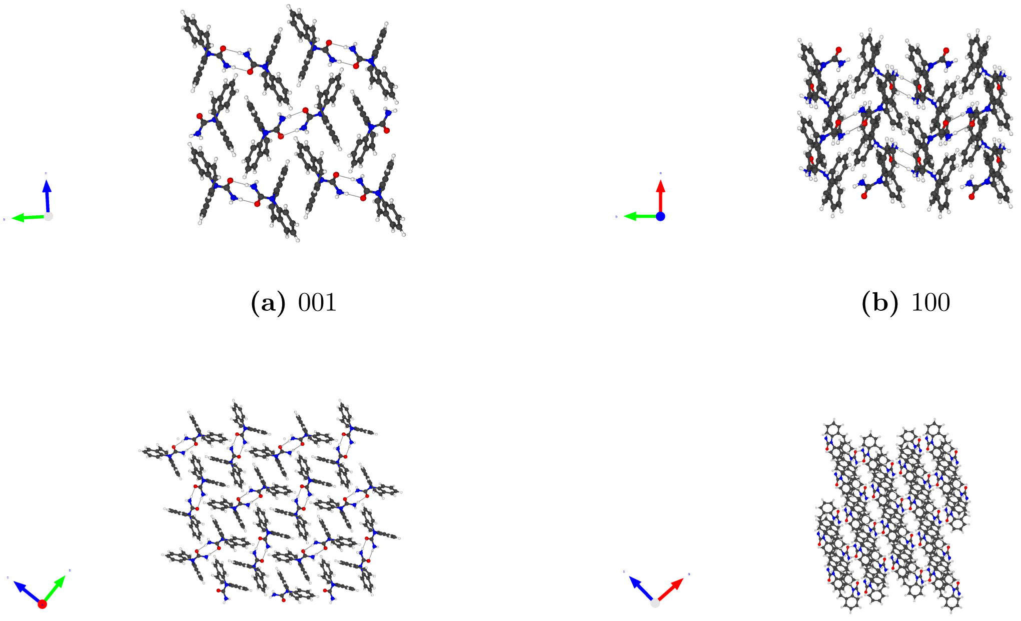

(a) 001

(b) 100

(c) 011

(d) 102

Figure S8: Additional surfaces modeled for Form III of CBZ (side view).

Stability of slabs

To evaluate the stability of the unrestrained slabs in water, each surface slab was run for 300 ns in isotropic NPT (v-rescale thermostat, Parrinello-Rahman barostat[18]) at 300 K and 1 bar, starting from the same box as for the metadynamics run, but without the additional molecule in solution. The box was equilibrated until the water density is close to the limit for TIP3P and the surfaces were inspected visually before and after the simulation.

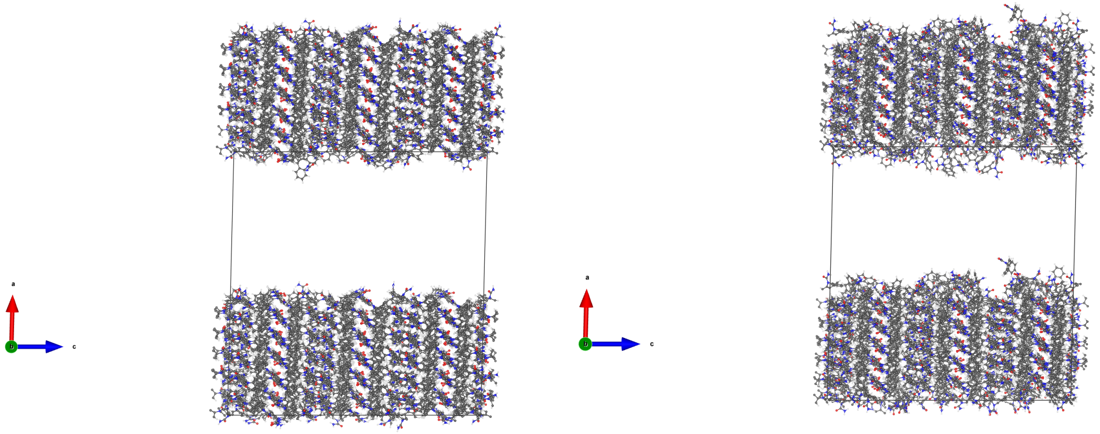

(a) before NPT **(b)** after NPT

Figure S9: Snapshots of Form I surface (100) before (a) and after (b) the NPT simulation. The periodic image in the x direction is shown on top for both pictures.

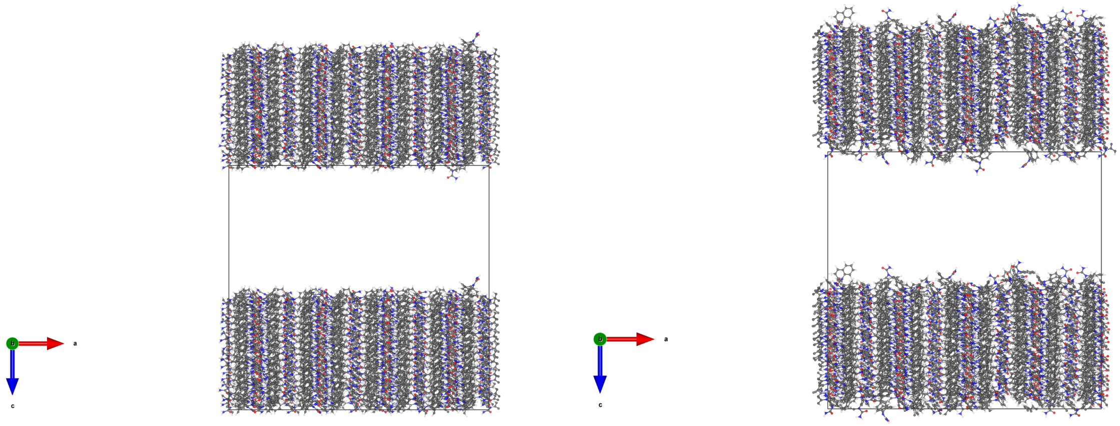

(a) before NPT **(b)** after NPT

Figure S10: Snapshots of Form II surface (001) before (a) and after (b) the NPT simulation. The periodic image in the z direction is shown on top for both pictures.

All forms show increased disorder at the surface, with the 011 surface of Form III even developing a step in the intact crystal structure. However, the crystal lattice is still visibly preserved in all surfaces, for the 010 surface of Form III, Form II and the dihydrate, the disorder at the surface is even minimal.

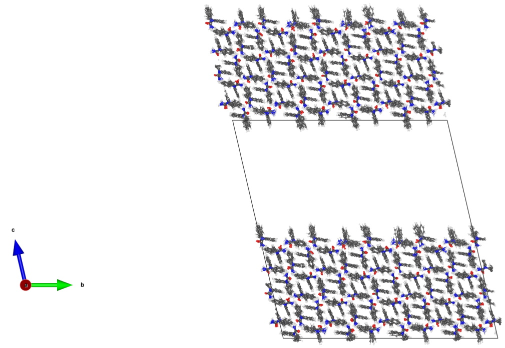

(a) before NPT

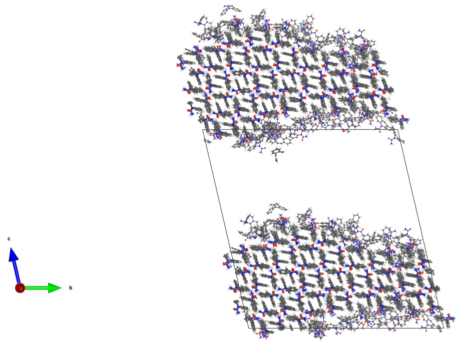

(b) after NPT

Figure S11: Snapshots of the 011 surface of Form III before (a) and after (b) the NPT simulation. The periodic image in the z direction is shown on top for both pictures.

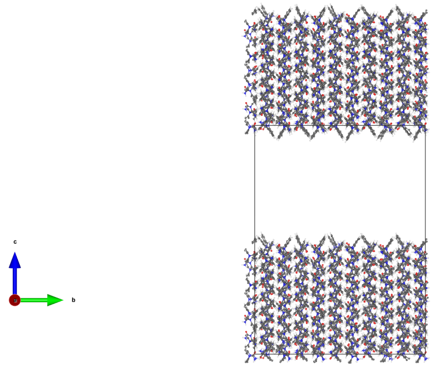

(a) before NPT

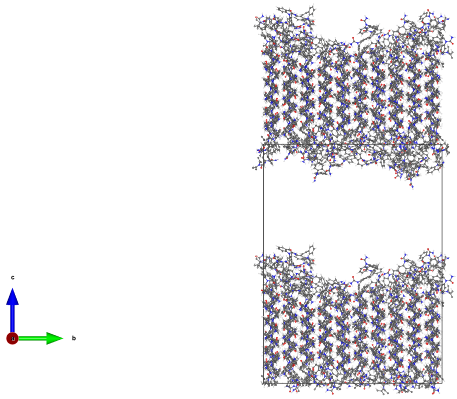

(b) after NPT

Figure S12: Snapshots of the 102 surface of Form III before (a) and after (b) the NPT simulation. The periodic image in the z direction is shown on top for both pictures.

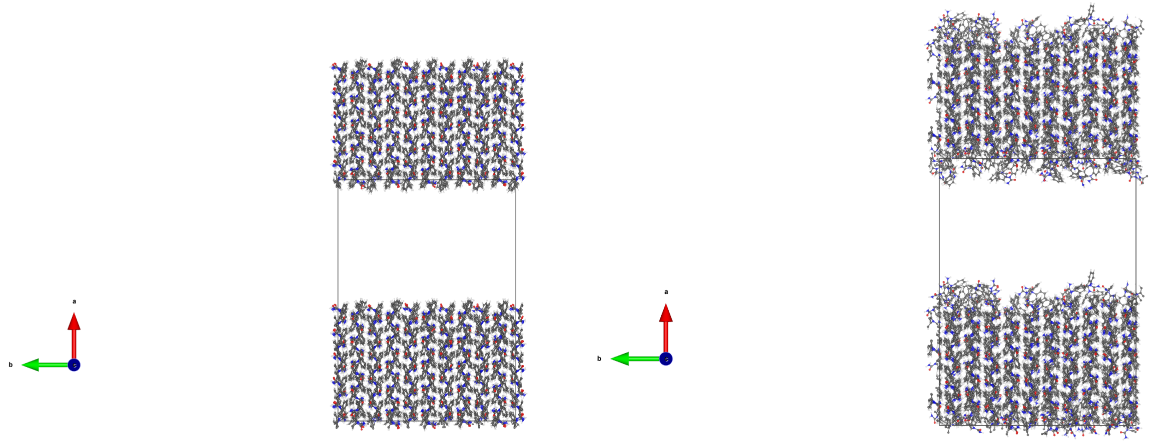

(a) before NPT **(b)** after NPT

Figure S13: Snapshots of the 100 surface of Form III before (a) and after (b) the NPT simulation. The periodic image in the x direction is shown on top for both pictures.

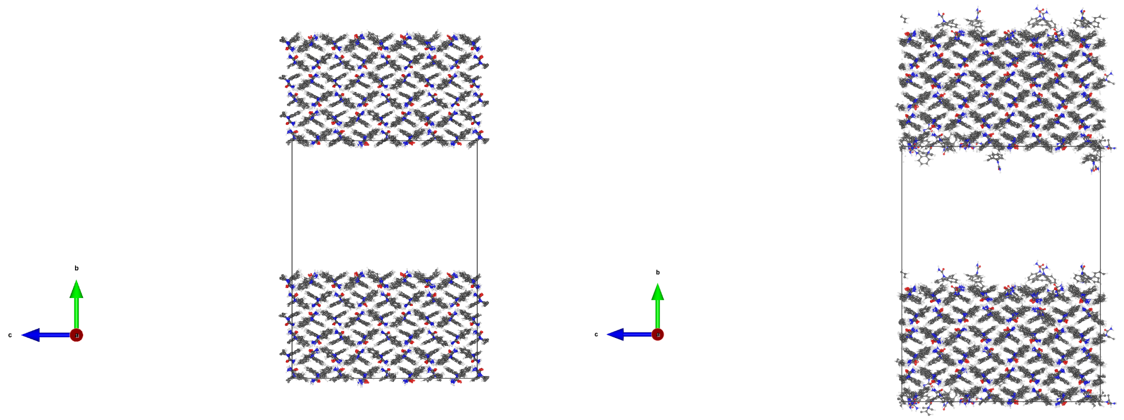

(a) before NPT **(b)** after NPT

Figure S14: Snapshots of the 010 surface of Form III before (a) and after (b) the NPT simulation. The periodic image in the y direction is shown on top for both pictures.

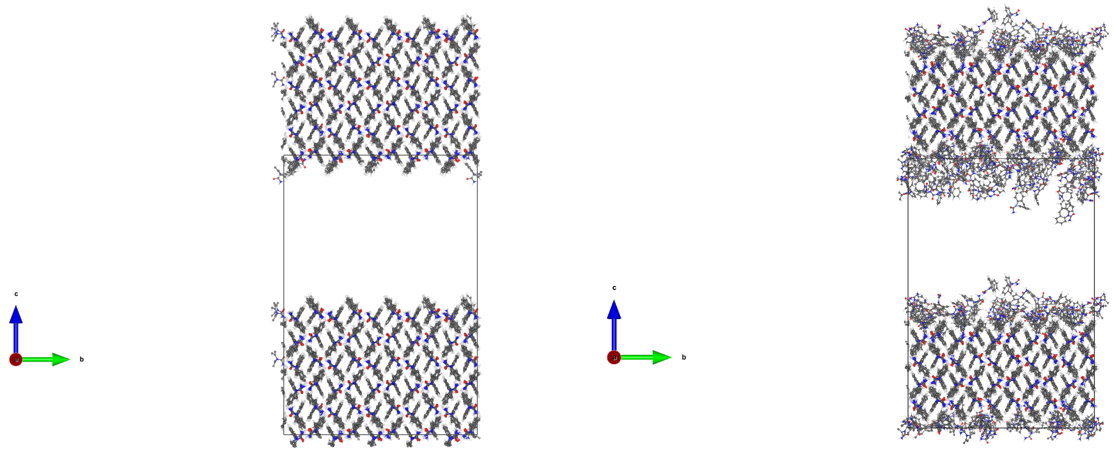

(a) before NPT **(b)** after NPT

Figure S15: Snapshots of the 001 surface of Form III before (a) and after (b) the NPT simulation. The periodic image in the z direction is shown on top for both pictures.

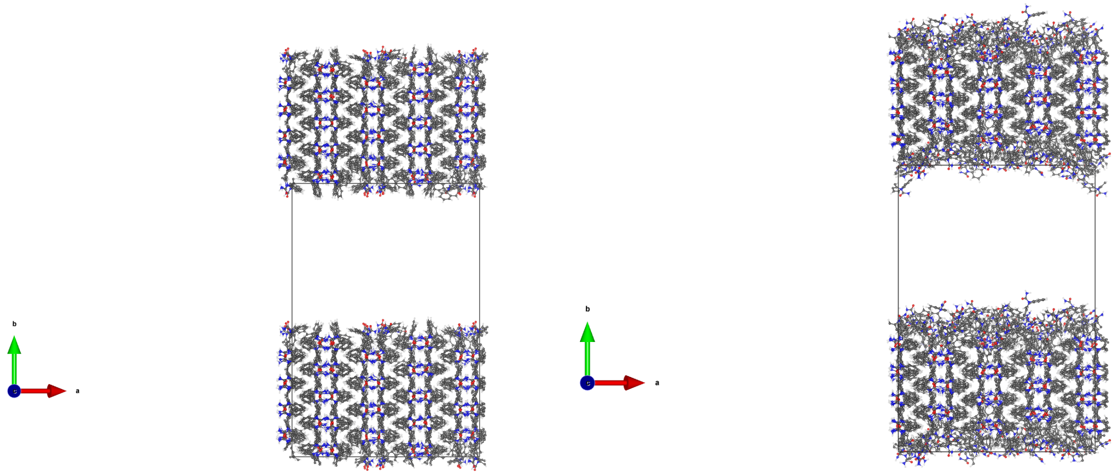

(a) before NPT **(b)** after NPT

Figure S16: Snapshots of Form IV surface (010) before (a) and after (b) the NPT simulation. The periodic image in the y direction is shown on top for both pictures.

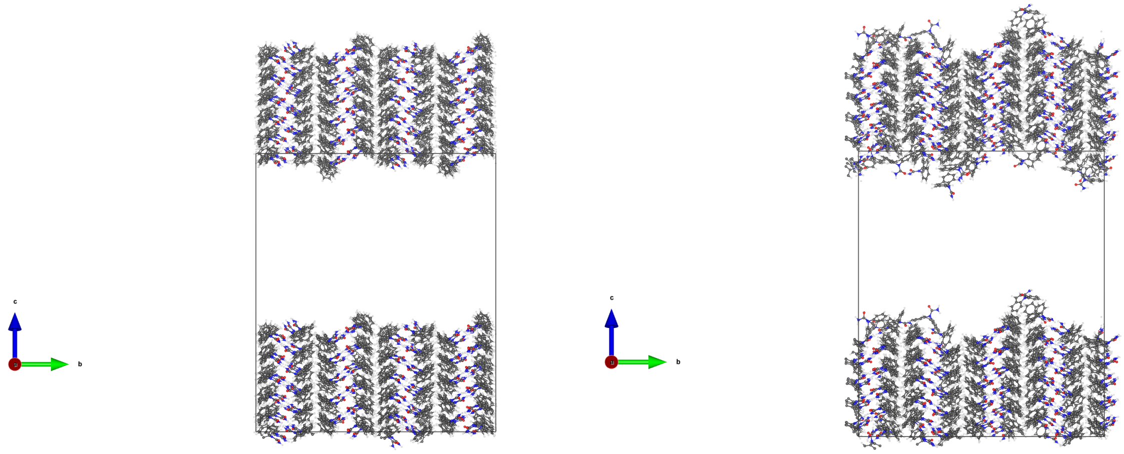

(a) before NPT **(b)** after NPT

Figure S17: Snapshots of the dihydrate surface (001) before (a) and after (b) the NPT simulation. The periodic image in the z direction is shown on top for both pictures.

Convergence and sampling of metadynamics simulations

Convergence of the metadynamics simulations is evaluated by comparing the negative of the stored grid (i.e. the applied bias averaged over the simulation) to the potential of mean force (PMF) obtained from force integration over the trajectory. Good matching of the two is seen for all forms, with minor deviations (see S18-S20). The completeness of the sampling is assessed by the coverage of the collective variable space during the simulation, i.e. how much of the surface separation distance is covered. All forms show good sampling between -0.5 and 1.5 nm, where the upper and lower walls were applied. Form I does not go below 0 nm, since the SSD is not defined as in the other forms, but as the minimum distance of the solute center of mass to any atom on the surface.

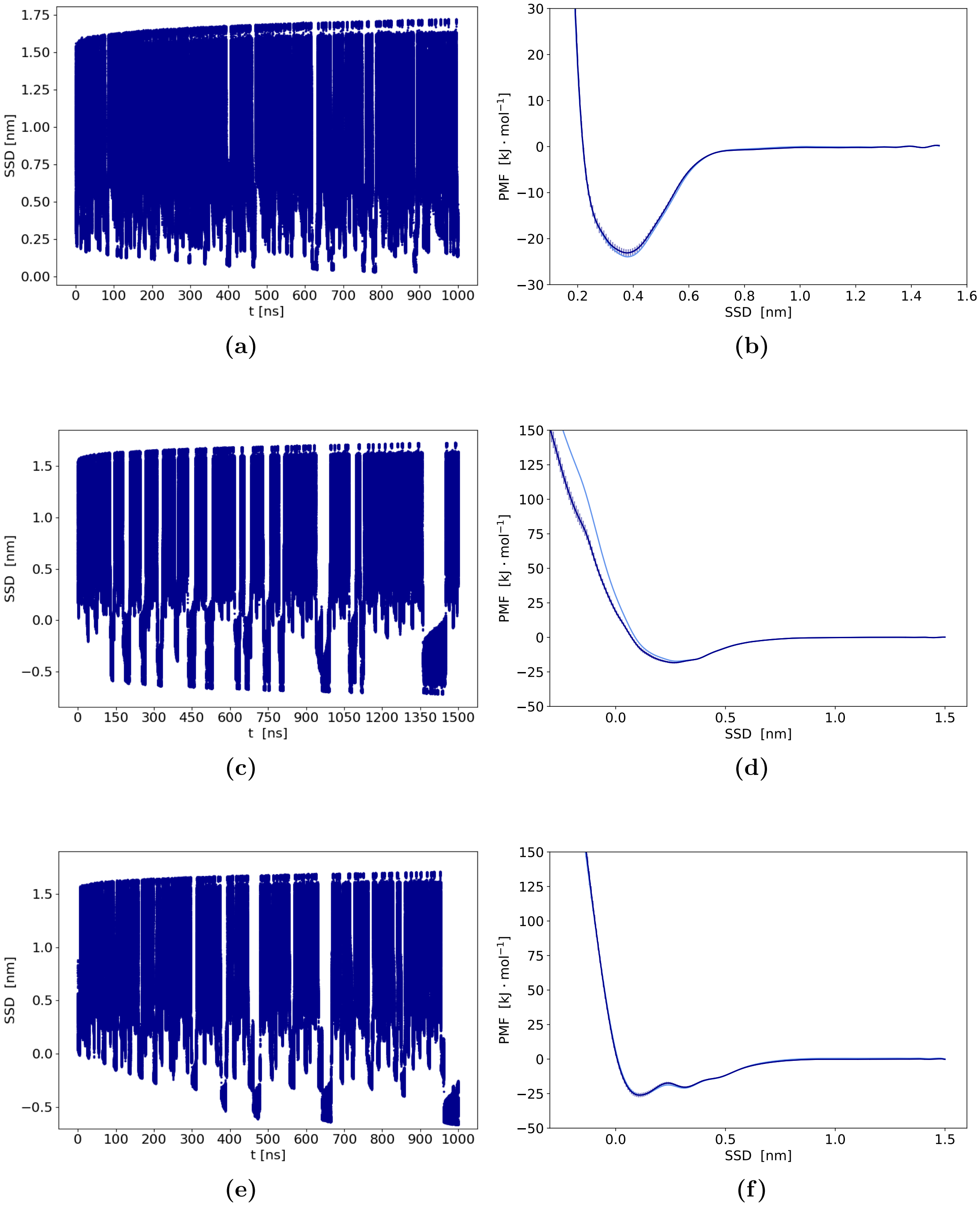

Figure S18: CV sampling and matching of grid bias stored by PLUMED (light blue) and PMF obtained by force integration (dark blue) for Form I (a,b), Form II (c, d) and the 011 surface of Form III (e, f).

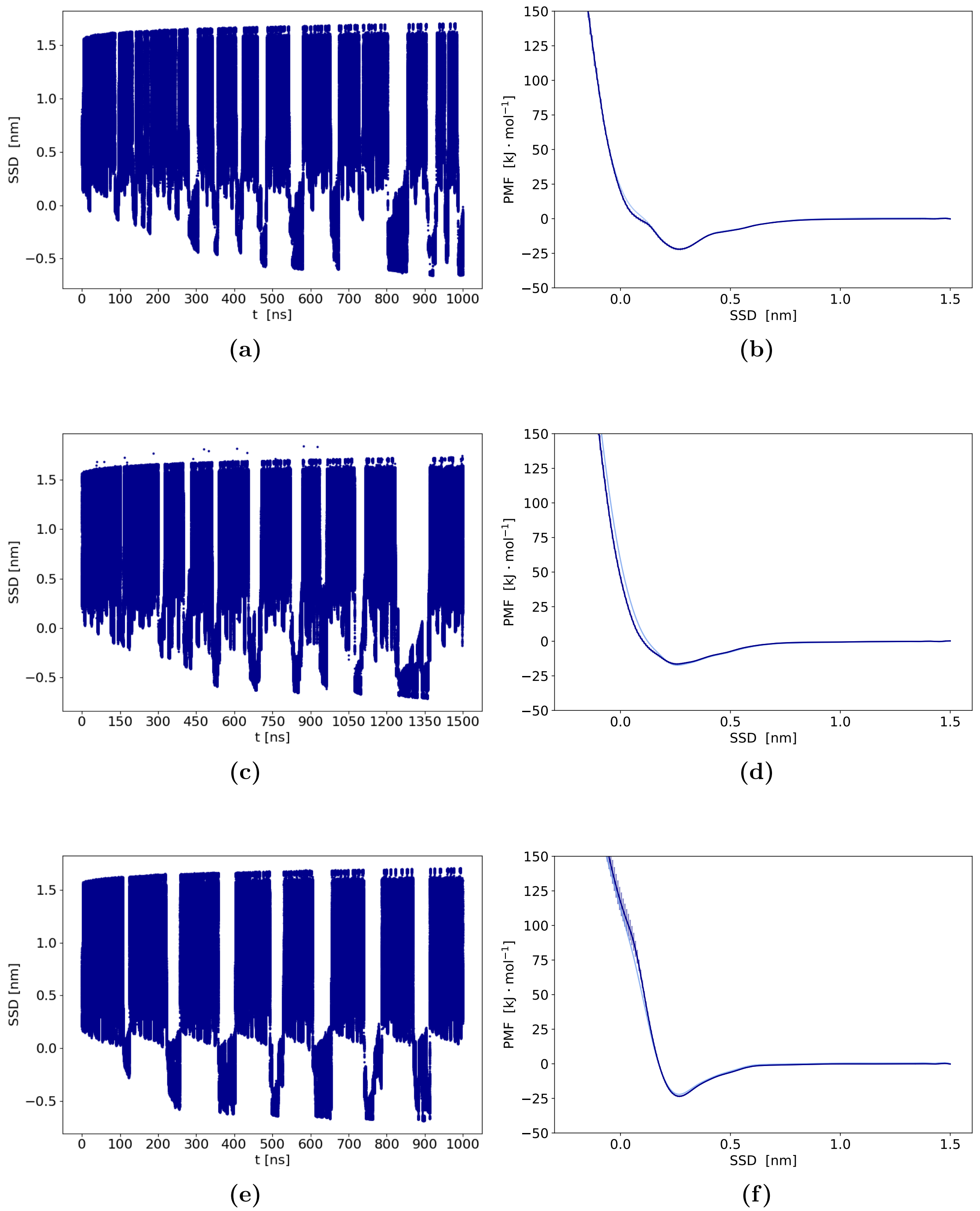

Figure S19: CV sampling and matching of grid bias stored by PLUMED (light blue) and PMF obtained by force integration (dark blue) for the 102 (a, b), the 100 (c, d) and the 010 (e, f) surfaces of Form III.

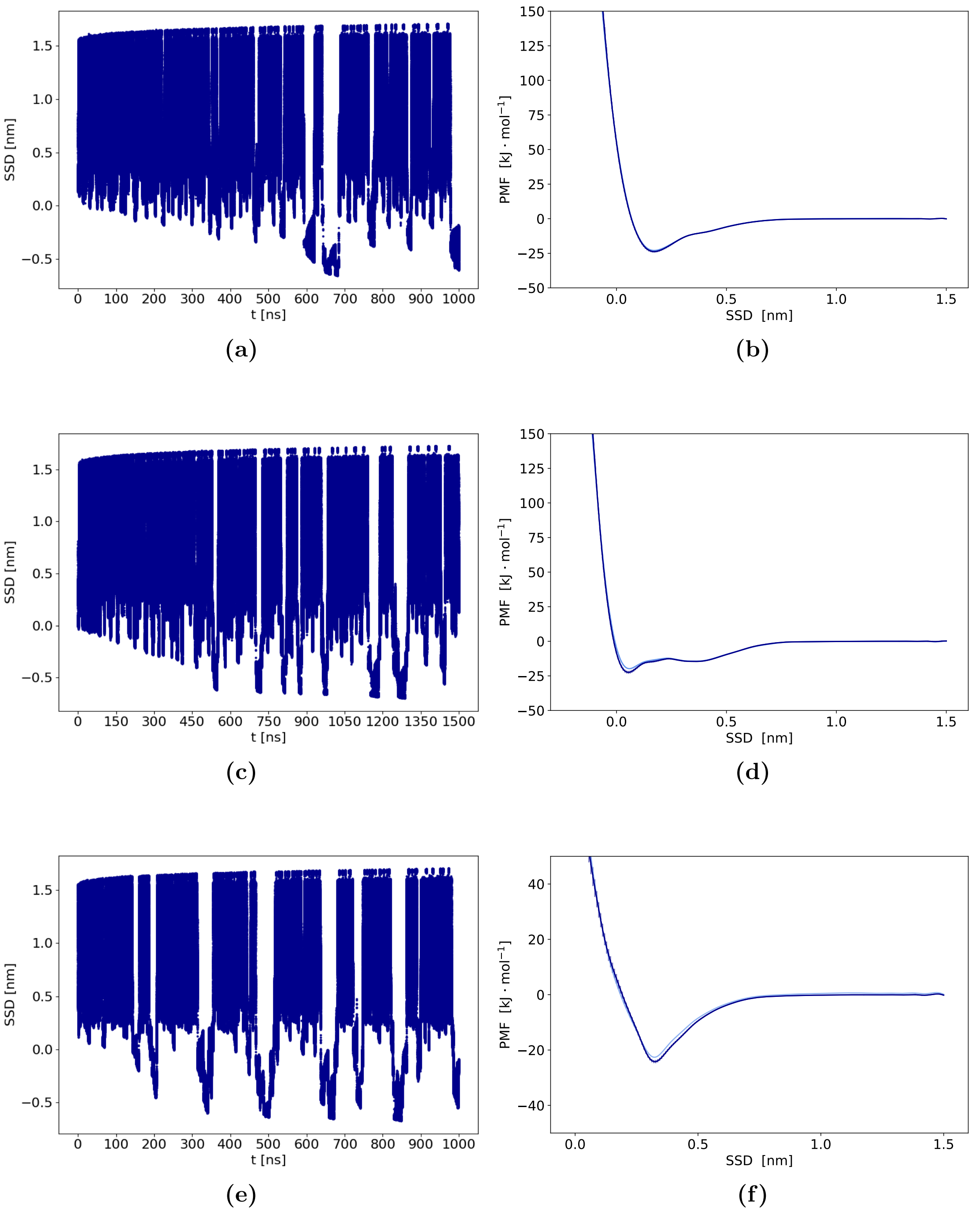

Figure S20: CV sampling and matching of grid bias stored by PLUMED (light blue) and PMF obtained by force integration (dark blue) for the 001 surface of Form III (a, b), Form IV (c, d) and the dihydrate (e, f).

Sampling of the accessible configuration space

To be able to compare between different simulations, one needs to ensure that the full volume of the solution region is sampled, i.e. the full configuration volume in the 'unbound' state. The good sampling of the SSD and the PMF approaching a constant value at larger distances is an indicator that the volume has been sampled adequately, but it was also calculated explicitly by computing the solvent-excluded surface of a PDB file consisting of all solute positions over the course of the simulation (more exactly all its center-of-mass positions, which were approximated by choosing the position of the N1 atom). This procedure is analogous to the calculation of the unbound volume in a paper by Bertazzo *et al.*,[19] and was performed using the NanoShaper software.[20] A key difference is that Bertazzo *et al.* only calculate the volume of the states defined as unbound and use this to correct their free energy of binding and obtain a standard free energy. In our case, we are calculating the full volume covered by the solute over the course of the simulation as a proxy for the coverage of the 'unbound' state, since we only want to ensure the correct entropy contribution in solution. The calculated volume was then compared to the available volume between the surface of the slab and the upper wall at 1.5 nm, correcting for the van der Waals radius of the nitrogen atom (about 1.8 Å). However, since the wall is a restraint and not a hard constraint, larger SSD values are possible, so the volume between the surface and the maximum SSD value was also calculated, again corrected for the van der Waals radius of the nitrogen atom. In principle, the available volume should be slightly higher than this, since the solute is not restrained at the surface, but 0.5 nm below it (lower wall), but most of the volume between the surface and the lower wall is already covered by molecules of the slab, so the contribution of this volume to the total volume is minor (depending on the structure of the slab, diffusion into the crystal structure is possible however). Table S2 shows the comparison of the calculated SES volume (V_{SES}), the volume between surface and upper wall ($V_{\mathrm{s-uw}}$) and the volume between the surface and the maximum SSD value ($V_{\mathrm{s-mcv}}$). Figures S21-S29 show the calculated volume compared to the positions of surface and upper/lower walls in

the simulation box. All results indicate a good coverage of the available volume, meaning the configuration space in solution is sampled fully and the correct entropy contribution is obtained.

Table S2: Solvent-excluded surface volume of the N1 atom of the solute during the simulation and available volume. $\boldsymbol{V}_{\mathbf{s-uw}}$ is the volume found between the surface and the upper wall (1.5 nm from the surface) and $\boldsymbol{V}_{\mathbf{s-mcv}}$ is the volume between the surface and the maximum SSD (both are corrected for the van der Waals radius of N1). All values are given in Å^3.

Form	Surface	$\boldsymbol{V}_{\mathbf{SES}}$	$\boldsymbol{V}_{\mathbf{s-uw}}$	$\boldsymbol{V}_{\mathbf{s-mcv}}$
I	100	88049.6	68803.6	77895.5
II	001	90176.7	73157.0	83085.5
III	100	64849.3	52142.3	62788.1
	010	71409.4	56229.9	65290.3
	001	61996.0	49440.7	55679.6
	011	75133.2	64661.9	72667.7
	102	50759.0	41173.8	46247.0
IV	010	60149.9	46992.3	53313.9
DH	001	49325.7	38853.1	43663.5

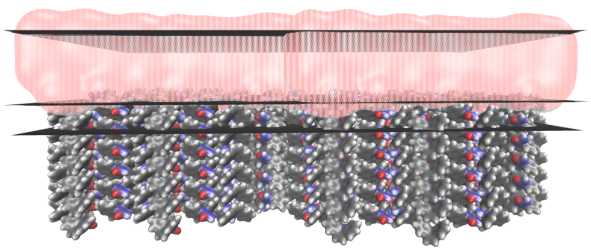

Figure S21: Solvent-excluded surface (pink) of the positions of the solute center of mass during the simulation for the Form I slab (one periodic copy shown). Black indicates the position of the surface (middle) and the lower and upper wall (bottom and top).

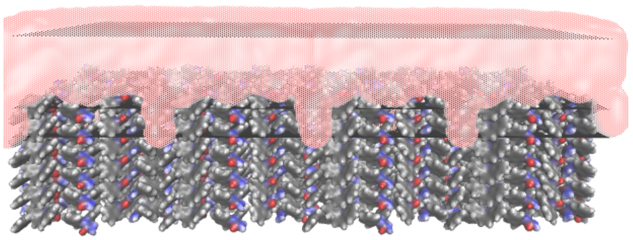

Figure S22: Solvent-excluded surface (pink) of the positions of the solute center of mass during the simulation for the Form I slab (one periodic copy shown). Black indicates the position of the surface (middle) and the lower and upper wall (bottom and top).

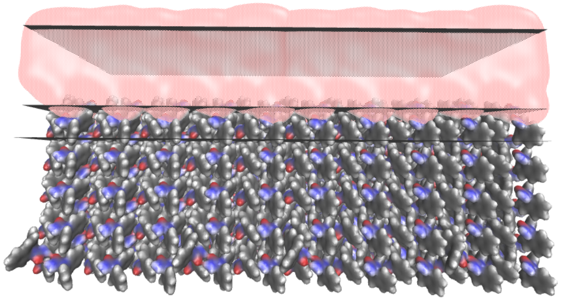

Figure S23: Solvent-excluded surface (pink) of the positions of the solute center of mass during the simulation for the Form III 100 slab (one periodic copy shown). Black indicates the position of the surface (middle) and the lower and upper wall (bottom and top).

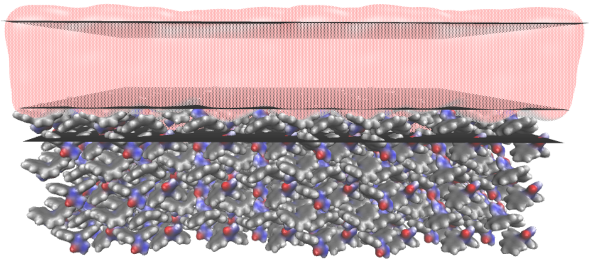

Figure S24: Solvent-excluded surface (pink) of the positions of the solute center of mass during the simulation for the Form III 010 slab (one periodic copy shown). Black indicates the position of the surface (middle) and the lower and upper wall (bottom and top).

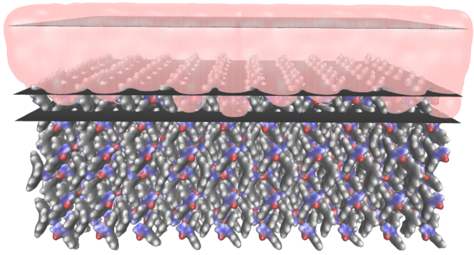

Figure S25: Solvent-excluded surface (pink) of the positions of the solute center of mass during the simulation for the Form III 001 slab (one periodic copy shown). Black indicates the position of the surface (middle) and the lower and upper wall (bottom and top).

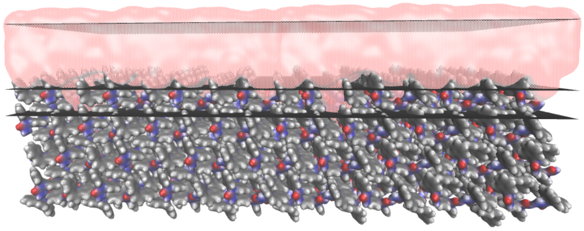

Figure S26: Solvent-excluded surface (pink) of the positions of the solute center of mass during the simulation for the Form III 011 slab (one periodic copy shown). Black indicates the position of the surface (middle) and the lower and upper wall (bottom and top).

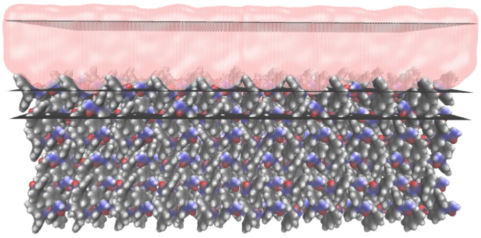

Figure S27: Solvent-excluded surface (pink) of the positions of the solute center of mass during the simulation for the Form III 102 slab (one periodic copy shown). Black indicates the position of the surface (middle) and the lower and upper wall (bottom and top).

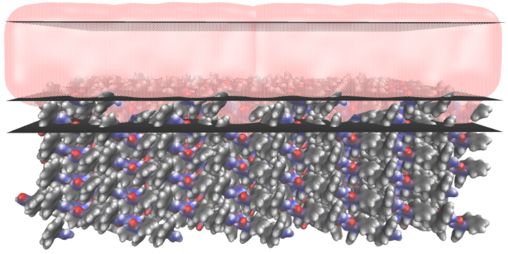

Figure S28: Solvent-excluded surface (pink) of the positions of the solute center of mass during the simulation for the Form IV slab (one periodic copy shown). Black indicates the position of the surface (middle) and the lower and upper wall (bottom and top).

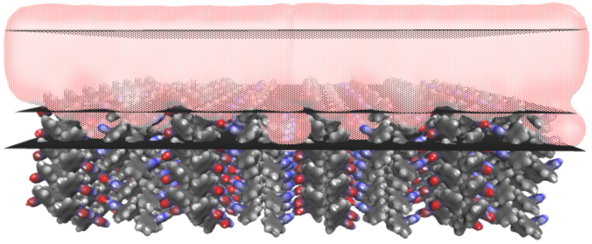

Figure S29: Solvent-excluded surface (pink) of the positions of the solute center of mass during the simulation for the dihydrate slab (one periodic copy shown). Black indicates the position of the surface (middle) and the lower and upper wall (bottom and top).

Crystal growth states

Finding states

The criteria to determine whether a state falls into crystal growth or not are described in the method section of the main paper. The distributions of the angles between the six-membered rings and the center-of-mass distances of those chosen from the angle distribution of the bound state are discussed there as well. Table S3 shows supplementary information for these states regarding the frequency of correct angle alignment and correct overall alignment in the bound data set.

Table S3: Results for crystal growth states in the bound data set for all forms.

Form	Surface	% of states at correct angle (of all states in the bound data set)	% of states at correct angle and correct COM distance	
			of all states in the bound data set	of all states at correct angle
I	100	5.722	4.289	79.766
II	001	9.993	8.294	86.420
III	100	8.593	6.649	79.030
	010	14.604	12.664	88.190
	001	6.707	5.407	82.104
	011	25.059	15.820	65.408
	102	6.352	4.651	75.726
IV	010	3.174	2.581	85.944
DH	001	5.878	4.824	83.667

Distribution of states and rescaled PMF integration

In fig.s S30 to S38, the distributions over the SSD of the found crystal growth states in the bound data set are shown, together with the rescaled PMF calculated from them. The part of the PMF used for integration of the free energy is marked as filled. As described in the methods section, the free energy change in the rescaled PMF is calculated from

$$\Delta G_{\mathrm{r}} \approx -k_{\mathrm{B}}T \ln \left(\frac{\sum_b \exp\{-\frac{W_r(\boldsymbol{s}_b)}{k_{\mathrm{B}}T}\}\Delta \boldsymbol{s}_b}{Z_{\mathrm{free}}} \right) \tag{3}$$

where the index b iterates over the part where the rescaled PMF is below zero and Z_{free} is the free volume. The error estimate is obtained by integrating first over $W_r + \Delta W_r$, then over $W_r - \Delta W_r$ and estimating the total error as the difference between the two divided by four (max-min/4).

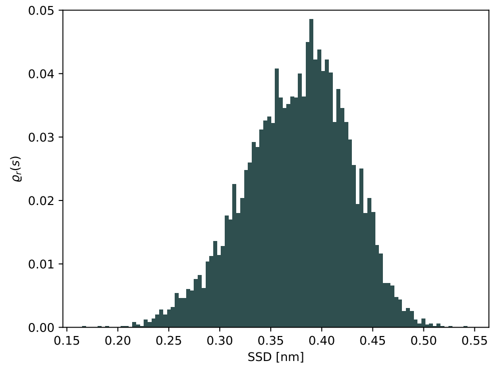

(a)

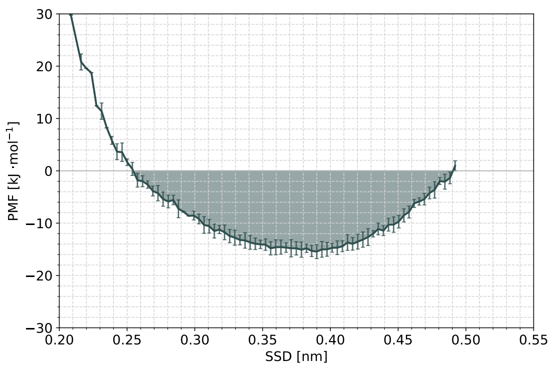

(b)

Figure S30: Distribution of found crystal growth states over the collective variable (a) and integration over rescaled PMF (b) in Form I.

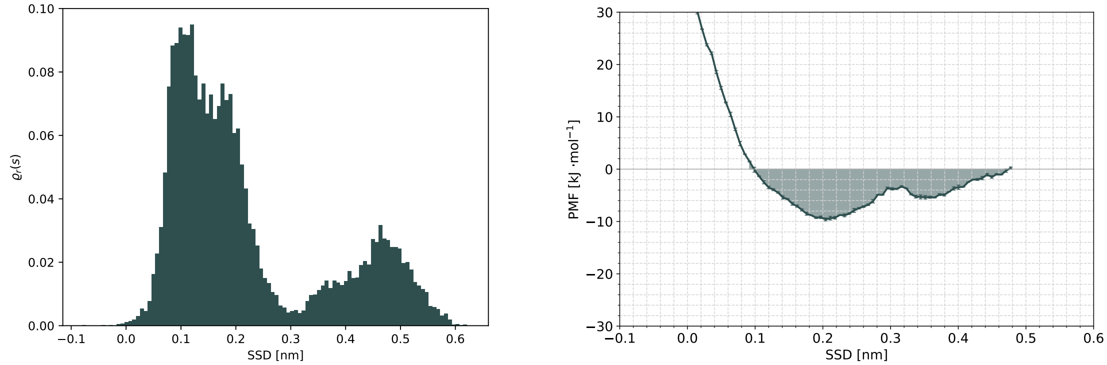

(a) **(b)**

Figure S31: Distribution of found crystal growth states over the collective variable (a) and integration over rescaled PMF(b) in Form II.

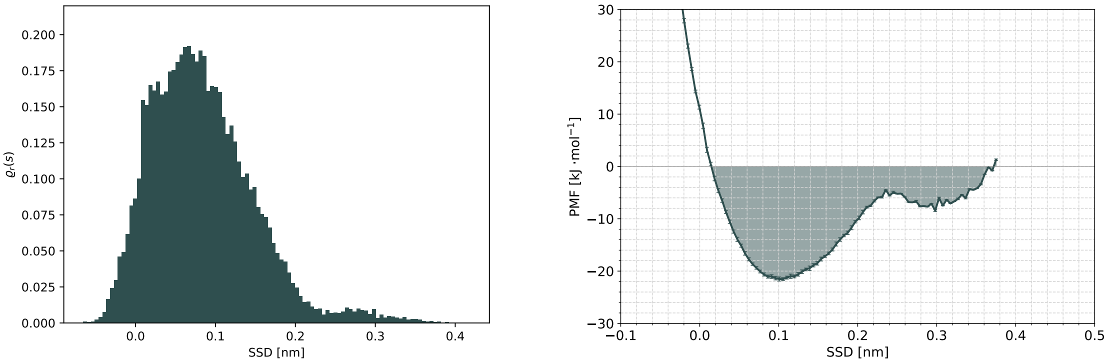

(a) **(b)**

Figure S32: Distribution of found crystal growth states over the collective variable (a) and integration over rescaled PMF (b) in Form III 011.

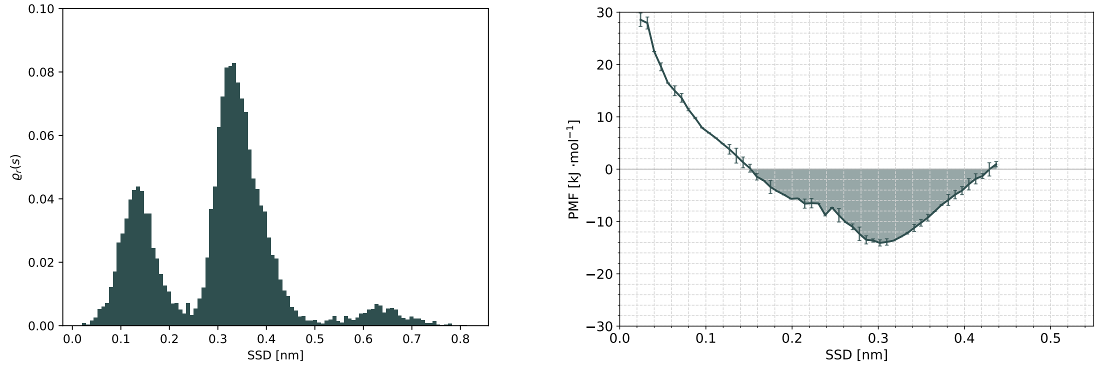

(a) **(b)**

Figure S33: Distribution of found crystal growth states over the collective variable (a) and integration over rescaled PMF (b) in Form III 102.

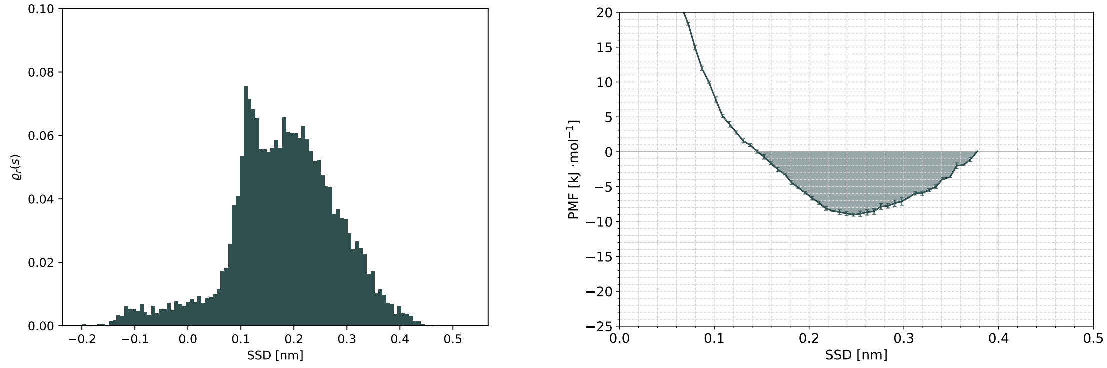

(a) **(b)**

Figure S34: Distribution of found crystal growth states over the collective variable (a) and integration over rescaled PMF (b) in Form III 100.

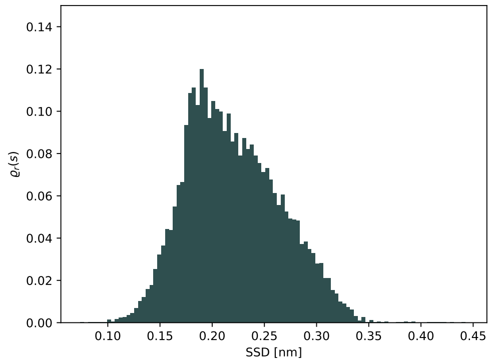

(a)

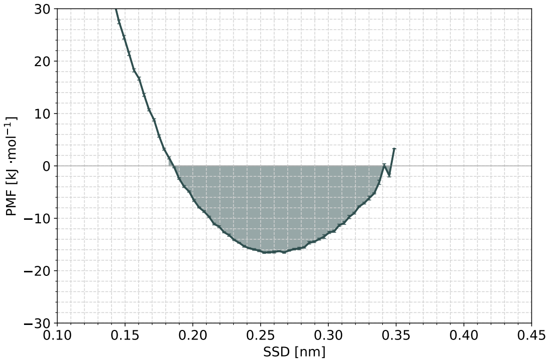

(b)

Figure S35: Distribution of found crystal growth states over the collective variable (a) and integration over rescaled PMF (b) in Form III 010.

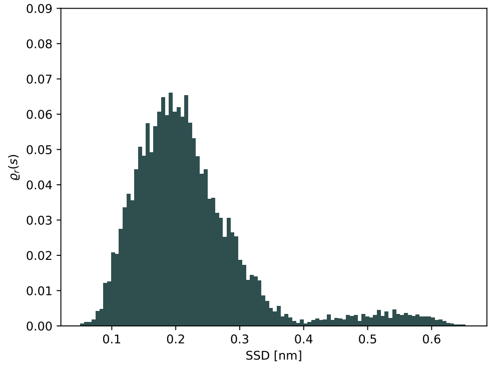

(a)

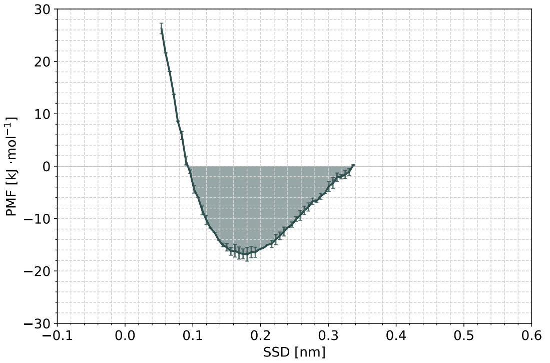

(b)

Figure S36: Distribution of found crystal growth states over the collective variable (a) and integration over rescaled PMF (b) in Form III 001.

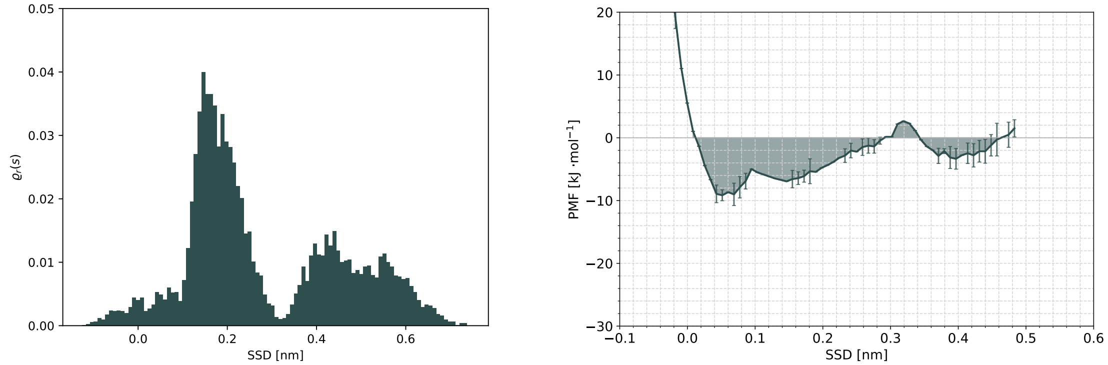

(a) **(b)**

Figure S37: Distribution of found crystal growth states over the collective variable (a) and integration over rescaled PMF (b) in Form IV.

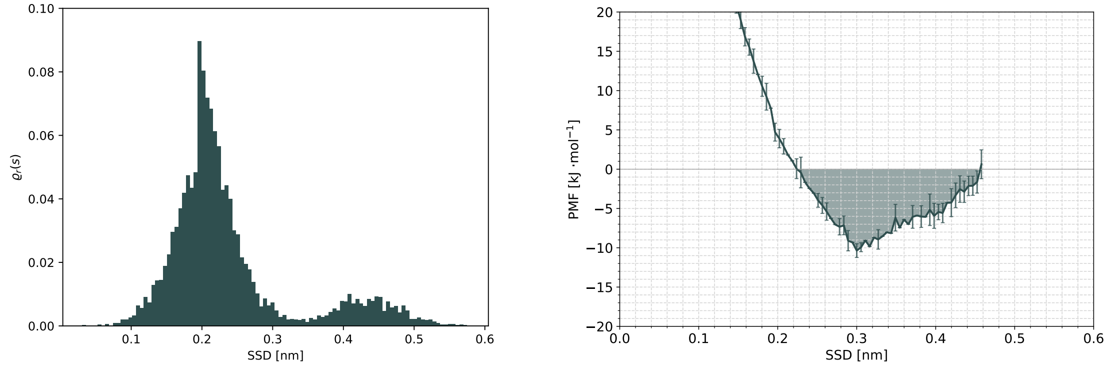

(a) **(b)**

Figure S38: Distribution of found crystal growth states over the collective variable (a) and integration over rescaled PMF (b) in DH.

Secondary maxima in the center-of-mass distance distributions

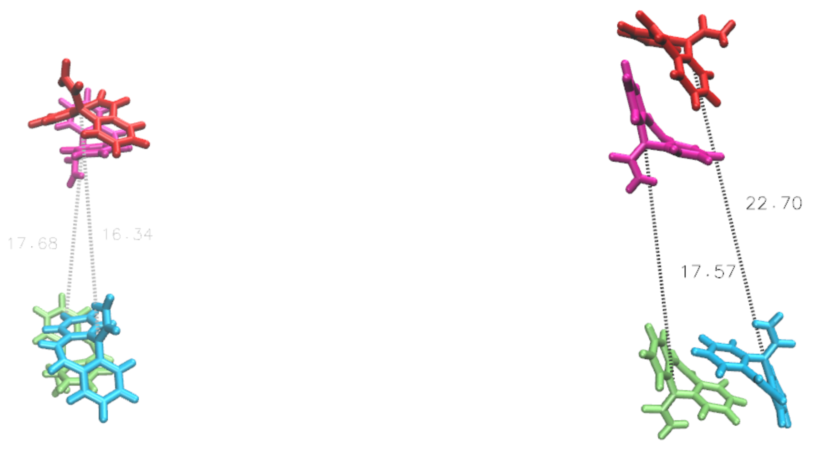

(a) **(b)**

Figure S39: Discarded states from the distribution of center-of-mass distances for all states with fully aligned angles in the bound data set for the 011 surface of Form III. (a) Orientation of solute (top) and nearest molecule in the slab (bottom) at the minor maximum. (b) Orientation of the solute (red) and the nearest molecule (blue) at the major maximum. Note that the solute should show the same orientation with respect to the pink molecule as the blue molecule does to the green. Dashed grey lines show the distance between N1 atoms as a stand-in for the distance between centers of the molecules.

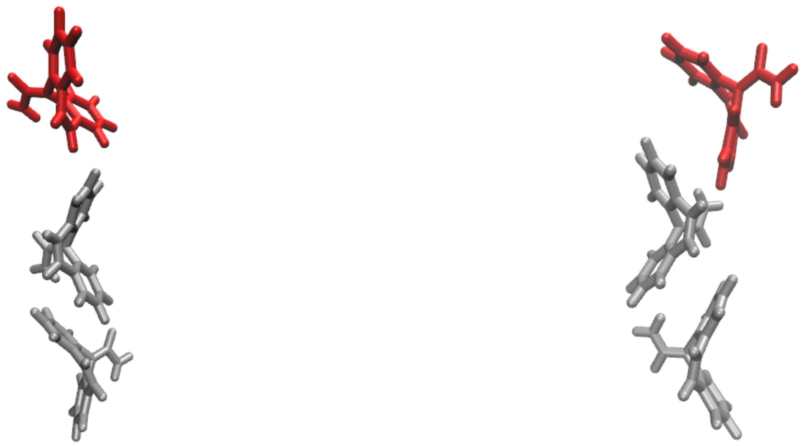

(a) **(b)**

Figure S40: Discarded 'satellite' states from the distribution of center-of-mass distances for all states with fully aligned angles in the bound data set for (a) the 001 surface of Form III and (b) the 102 surface of Form III. Note that the solute, marked in red, should align with the grey molecule at the bottom.

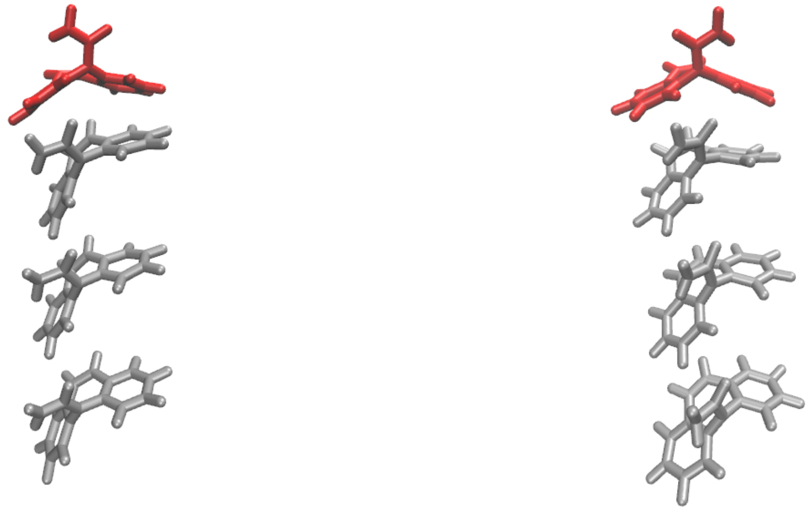

(a) **(b)**

Figure S41: States from the two secondary maxima in Form IV. In both (a) and (b), the solute in red shows good alignment with the grey molecules in the repeating layers of the slab, so these were counted as aligned.

Alternate states

As described in the method section of the main paper, there are three distinct ways the molecule can be oriented at the surface (cf. fig. S42).

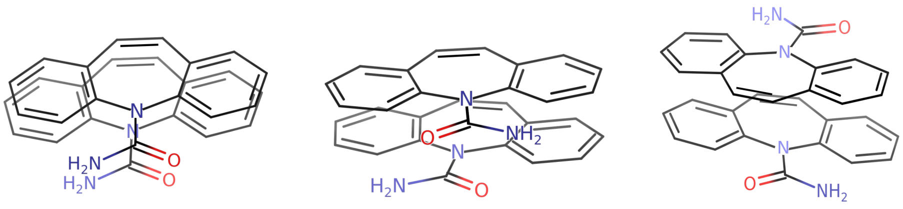

(a) (i) **(b)** (ii) **(c)** (iii)

Figure S42: The three orientations of the solute molecule at the surface in alignment with the crystal structure: (i) full alignment of all atoms in the molecule of the slab to the solute, (ii) full alignment of the ring system, but the amide group in the solute is rotated 180° with respect to the amide group at the surface, (iii) the ring system in the solute is rotated 180° with respect to the ring system in the slab.

How much they contribute to the total number of crystal growth states was evaluated by defining two more vectors: a vector connecting the C7 atom in the seven-membered ring to the N1 atom that would indicate a rotation of the ring system (see fig. S43 a) and a vector connecting the C15 atom in the amide group to the O1 atom to indicate rotation of this group (see fig. S43 b).

The distribution of angles of both vectors in the crystal growth states were evaluated and are shown in the following pages. For the C7-N1 an angle of about $0 - 50°$ can be counted as full alignment of the ring system and angles $> 100°$ are counted as rotated states. For the C15-O1 vector fully aligned states fall around $0 - 50°$ and rotated states are $> 90°$. Plotting the joint angle distribution introduces one more orientation, where both the ring system and the amide are rotated with respect to the orientation of the molecule in the slab. This

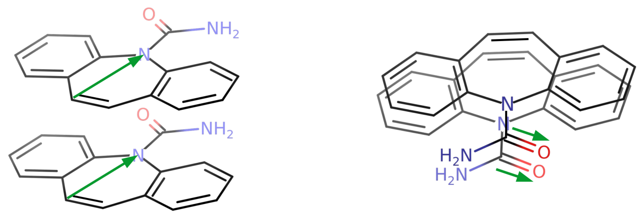

(a) **(b)**

Figure S43: Additional vectors evaluated to characterize the orientation of the solute molecule on the surface, here shown in full alignment. (a) C7-N1 vector used to identify whether the ring system is rotated (b) C15-O1 vector used to identify the orientation of the amide group.

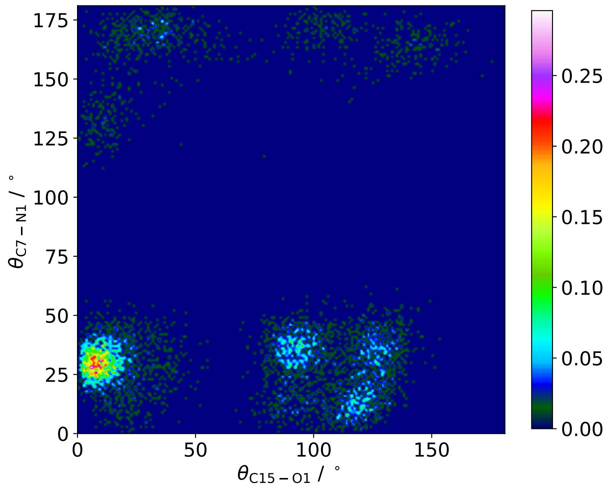

Figure S44: Probability distribution of aligned states with respect to the vectors defined in fig. S43 for Form I.

configuration is simply regarded as orientation (iii), since it does not matter what orientation the amide has in this one, since it is not in the vicinity of another amide group.

Form I shows most states in full alignment (bottom left corner), with a minor contribution from state (ii), where the amide group is rotated (bottom right). Very few states are in an orientation where the ring system is rotated (upper half of the plot) and both the (ii) and (iii) peaks are very scattered. Form II shows an almost even partitioning of states between the different orientations, with slightly more in orientation (iii). All peaks are quite sharp,

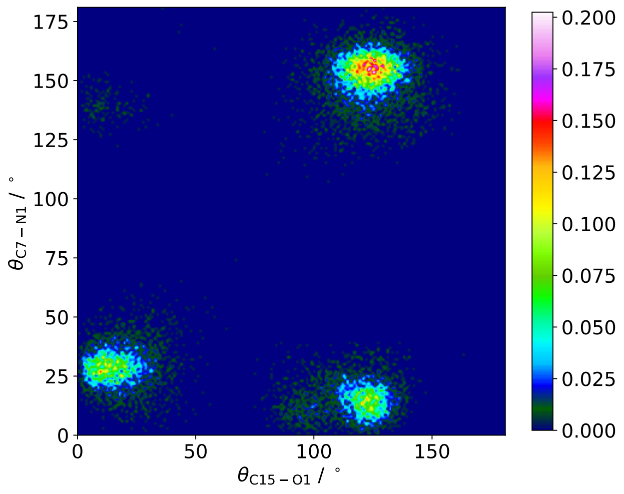

Figure S45: Probability distribution of aligned states with respect to the vectors defined in fig. S43 for Form II.

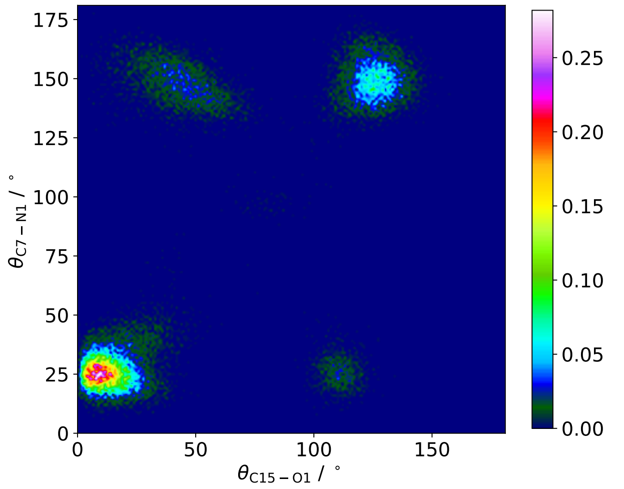

Figure S46: Probability distribution of aligned states with respect to the vectors defined in fig. S43 for Form III 011.

with low spread.

The 011 surface of Form III has most states in full alignment (bottom left), but a considerable

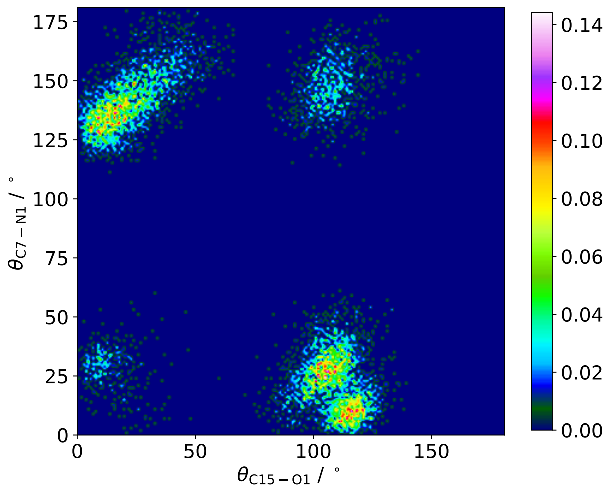

Figure S47: Probability distribution of aligned states with respect to the vectors defined in fig. S43 for Form III 102.

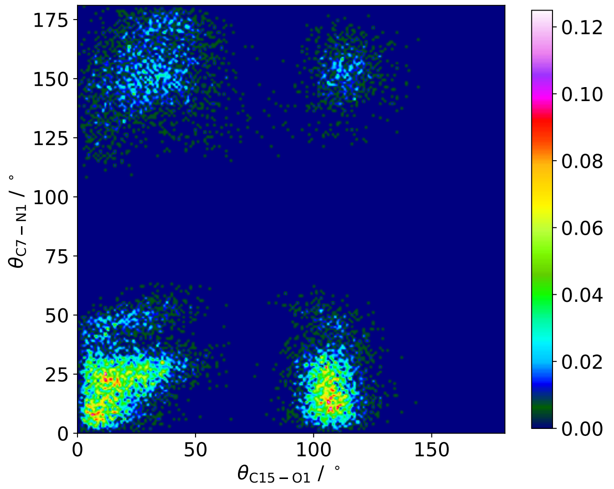

Figure S48: Probability distribution of aligned states with respect to the vectors defined in fig. S43 for Form III 100.

amount also in orientation (iii) in the upper half of the plot; peaks are even sharper than in Form II. The 102 surface has most of the states not in full alignment, but in either (ii) or

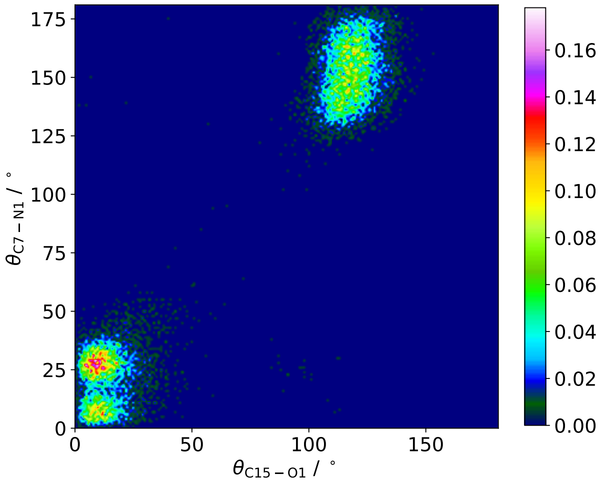

Figure S49: Probability distribution of aligned states with respect to the vectors defined in fig. S43 for Form III 010.

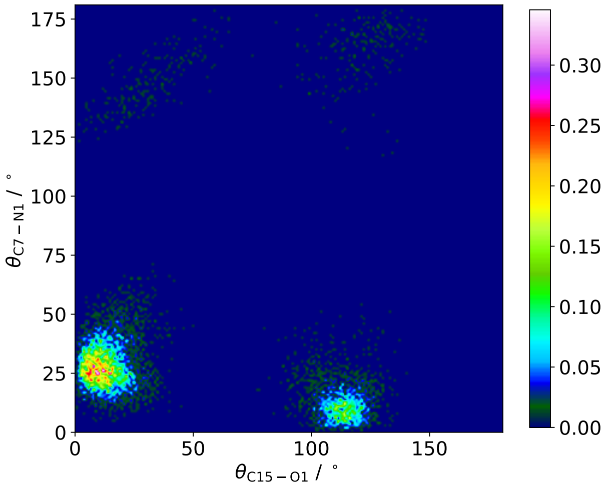

Figure S50: Probability distribution of aligned states with respect to the vectors defined in fig. S43 for Form III 001.

(iii), although some states do appear in full alignment. All peaks show a higher spread than in the 011 surface. The 100 surface of Form III has most states in either full alignment or

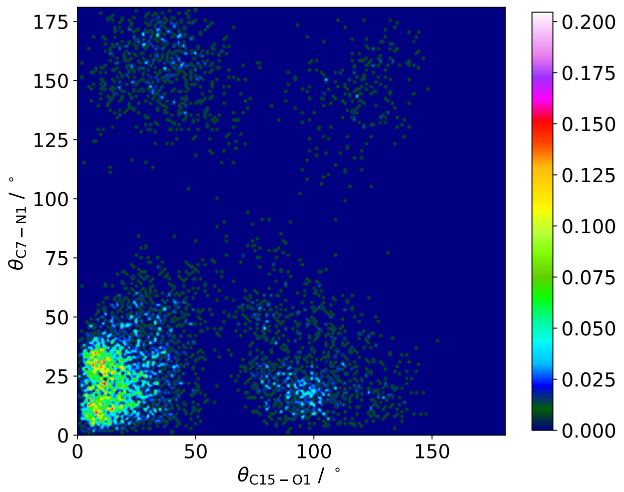

Figure S51: Probability distribution of aligned states with respect to the vectors defined in fig. S43 for Form IV.

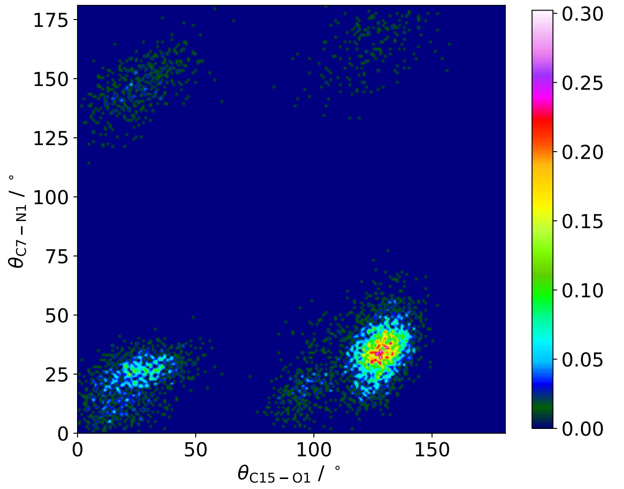

Figure S52: Probability distribution of aligned states with respect to the vectors defined in fig. S43 for DH.

with the amide group rotated (ii). Few states are found in orientation (iii) in the upper half of the plot. All peaks show some spread in both directions of the axes. For the 010 surface,

there are almost no states at all in orientation (ii), but they are split between full alignment and orientation (iii), with the full alignment slightly favored. The peaks show some spread in y direction (C7-N1 vector), but not much in x (C15-O1 vector).

The 001 surface of Form III on the other hand shows almost no states with the ring system rotated (iii), but most of them in full alignment or with the amide group rotated (ii), again favoring the former slightly. The spread of peaks is low. Form IV shows a preference for fully aligned states, with only very minor contributions from (ii) and (iii), but the spread of peaks is large, indicating that the solute has a lot more conformational flexibility. In the dihydrate (DH), orientation (ii) seems to be favored, with a second peak showing for the full alignment. The spread of peaks is moderate.

References

(1) Hunter, C. A.; McCabe, J. F.; Spitaleri, A. Solvent effects of the structures of prenucleation aggregates of carbamazepine. *CrystEngComm* **2012**, *14*, 7115–7117.

(2) Jorgensen, W. L.; Maxwell, D. S.; Tirado-Rives, J. Development and Testing of the OPLS All-Atom Force Field on Conformational Energetics and Properties of Organic Liquids. *Journal of the American Chemical Society* **1996**, *118*, 11225–11236.

(3) Wang, J.; Wolf, R. M.; Caldwell, J. W.; Kollman, P. A.; Case, D. A. Development and testing of a general amber force field. *Journal of Computational Chemistry* **2004**, *25*, 1157–1174.

(4) Frisch, M. J. et al. Gaussian 16 Revision C.01. 2016; Gaussian Inc. Wallingford CT.

(5) Sousa da Silva, A. W.; Vranken, W. F. ACPYPE - AnteChamber PYthon Parser interfacE. *BMC Research Notes* **2012**, *5*, 367.

(6) Fan, S.; Iorga, B. I.; Beckstein, O. Prediction of octanol-water partition coefficients for the SAMPL6-log P molecules using molecular dynamics simulations with OPLS-AA, AMBER and CHARMM force fields. *Journal of Computer-Aided Molecular Design* **2020**, *34*, 543–560.

(7) Šegatin, N.; Klofutar, C. Thermodynamics of the Solubility of Water in 1-Hexanol, 1-Octanol, 1-Decanol, and Cyclohexanol. *Monatshefte für Chemie / Chemical Monthly* **2004**, *135*, 241–248.

(8) Zangi, R. Refinement of the OPLSAA Force-Field for Liquid Alcohols. *ACS Omega* **2018**, *3*, 18089–18099.

(9) Wang, J.; Wang, W.; Kollman, P. A.; Case, D. A. Automatic atom type and bond type perception in molecular mechanical calculations. *Journal of Molecular Graphics and Modelling* **2006**, *25*, 247–260.

(10) Larsen, A. H. et al. The atomic simulation environment—a Python library for working with atoms. *Journal of Physics: Condensed Matter* **2017**, *29*, 273002.

(11) Dolomanov, O. V.; Bourhis, L. J.; Gildea, R. J.; Howard, J. a. K.; Puschmann, H. OLEX2: a complete structure solution, refinement and analysis program. *Journal of Applied Crystallography* **2009**, *42*, 339–341.

(12) Bussi, G.; Donadio, D.; Parrinello, M. Canonical sampling through velocity rescaling. *The Journal of Chemical Physics* **2007**, *126*, 014101.

(13) Berendsen, H. J. C.; Postma, J. P. M.; van Gunsteren, W. F.; DiNola, A.; Haak, J. R. Molecular dynamics with coupling to an external bath. *The Journal of Chemical Physics* **1984**, *81*, 3684–3690.

(14) Grzesiak, A. L.; Lang, M.; Kim, K.; Matzger, A. J. Comparison of the Four Anhydrous Polymorphs of Carbamazepine and the Crystal Structure of Form I. *Journal of Pharmaceutical Sciences* **2003**, *92*, 2260–2271.

(15) O'Mahony, M.; C. Seaton, C.; M. Croker, D.; Veesler, S.; C. Rasmuson, A.; K. Hodnett, B. Investigating the dissolution of the metastable triclinic polymorph of carbamazepine using in situ microscopy. *CrystEngComm* **2014**, *16*, 4133–4141, Publisher: Royal Society of Chemistry.

(16) Schneider-Rauber, G.; Bond, A. D.; Ho, R.; Nere, N.; Bordawekar, S.; Sheikh, A. Y.; Jones, W. Effect of Solution Composition on the Crystallization of Multicomponent Forms of Carbamazepine beyond Crystal Form and Shape: Surface as a Source of Diversity in the Solid-Form Landscape. *Crystal Growth & Design* **2021**, *21*, 52–64.

(17) Pronk, S.; Páll, S.; Schulz, R.; Larsson, P.; Bjelkmar, P.; Apostolov, R.; Shirts, M. R.; Smith, J. C.; Kasson, P. M.; van der Spoel, D.; Hess, B.; Lindahl, E. GROMACS 4.5: a high-throughput and highly parallel open source molecular simulation toolkit. *Bioinformatics* **2013**, *29*, 845–854.

(18) Parrinello, M.; Rahman, A. Crystal Structure and Pair Potentials: A Molecular-Dynamics Study. *Physical Review Letters* **1980**, *45*, 1196–1199.

(19) Bertazzo, M.; Gobbo, D.; Decherchi, S.; Cavalli, A. Machine Learning and Enhanced Sampling Simulations for Computing the Potential of Mean Force and Standard Binding Free Energy. *Journal of Chemical Theory and Computation* **2021**, *17*, 5287–5300.

(20) Decherchi, S.; Rocchia, W. A general and Robust Ray-Casting-Based Algorithm for Triangulating Surfaces at the Nanoscale. *PLOS ONE* **2013**, *8*, e59744.
